# Acute Endocrine Alterations After Traumatic Brain Injury and 6‐Month Functional Outcome

**DOI:** 10.1002/acn3.70570

**Published:** 2026-10-29

**Authors:** Biao Xu, Peng Guo, Rui Pan, Zhenghao Wang, Jingchao Zhou, Pengcheng Zhang, Xingzhe Zhang, Qinghua Zhang, Haibin Ma, Zhanfeng Niu

**Affiliations:** ^1^ The First Clinical Medical College of Ningxia Medical University Yinchuan Ningxia China; ^2^ Key Laboratory of Ningxia Stem Cell and Regenerative Medicine, Institute of Medical Sciences General Hospital of Ningxia Medical University Yinchuan China; ^3^ The 904th Hospital of the Joint Logistic Support Force of PLA Wuxi Jiangsu China; ^4^ Department of Neurosurgery General Hospital of Ningxia Medical University Yinchuan Ningxia China

**Keywords:** endocrine alterations, functional outcome, inflammation, thyroid hormones, traumatic brain injury

## Abstract

**Objective:**

Acute multi‐axis endocrine alterations after traumatic brain injury (TBI) remain incompletely characterized. This study aimed to examine their temporal patterns and explore their associations with 6‐month functional outcome and early inflammatory markers.

**Methods:**

We conducted a single‐center prospective observational cohort study of hospitalized adults with traumatic brain injury. Among 130 enrolled patients, 125 with complete 6‐month Glasgow Outcome Scale‐Extended follow‐up were included. Blood samples were collected on Day 1, 7, and 14 after injury to assess hypothalamic–pituitary–adrenal, thyroid, gonadal, and growth hormone axes. Day‐1 inflammatory markers were measured. Associations of individual biomarkers with unfavorable 6‐month outcome were evaluated using separate Firth penalized logistic regression models adjusted for admission Glasgow Coma Scale (GCS).

**Results:**

Serum cortisol levels at 08:00 and 16:00 decreased significantly over time (Kendall's *W* = 0.205 [95% CI 0.085–0.388] and 0.239 [95% CI 0.126–0.408], respectively; both *p* < 0.001), whereas triiodothyronine, free triiodothyronine, and thyroid‐stimulating hormone increased during the acute phase. Patients with unfavorable 6‐month outcome had higher day‐1 interleukin‐6, neutrophil count, and procalcitonin levels and lower day‐1 triiodothyronine and free triiodothyronine levels than those with favorable outcome. After adjustment for admission GCS in separate Firth models, interleukin‐6, white blood cell count, neutrophil count, procalcitonin, day‐1 triiodothyronine, and day‐1 free triiodothyronine were not significantly associated with unfavorable 6‐month outcome.

**Interpretation:**

Endocrine alterations after acute TBI were heterogeneous across injury severity groups and time points. Although selected day‐1 inflammatory and thyroid‐axis measures differed between outcome groups, their associations with unfavorable 6‐month functional outcome were not statistically significant after adjustment for admission GCS. These outcome‐related findings require validation in larger cohorts.

## 1 Introduction

Traumatic brain injury (TBI) remains a major cause of death and long‐term disability worldwide and continues to impose a substantial clinical, societal, and economic burden [1]. Increasing evidence suggests that, beyond the primary mechanical insult, the early course after TBI is shaped by a complex cascade of secondary injury processes involving neuroinflammation, endocrine dysregulation, and metabolic stress [2, 3, 4].

Previous studies suggest that TBI may disrupt multiple hypothalamic–pituitary–target gland axes, resulting in variable abnormalities involving the hypothalamic–pituitary–adrenal, thyroid, growth hormone, and gonadal systems [2, 5, 6, 7]. At the same time, growing evidence indicates that some endocrine abnormalities may be transient in the acute phase, whereas others may persist or become clinically evident only later during follow‐up, complicating the interpretation of single‐time‐point measurements [8, 9]. However, the available evidence remains inconsistent, partly because of marked heterogeneity in study populations, sampling windows, hormone panels, and outcome definitions [2, 4, 8, 10].

Thyroid‐axis dysfunction appears to be of particular interest among the endocrine abnormalities reported after TBI [11, 12]. Decreased concentrations of triiodothyronine (T3) and free triiodothyronine (FT3) are commonly regarded as manifestations of non‐thyroidal illness syndrome in acute critical illness and may reflect systemic stress, altered energy metabolism, and disrupted physiological homeostasis after injury [13]. In patients with acute stroke, low T3 status has been associated with greater disease severity and poorer clinical outcomes [14]. Endocrine alterations after TBI may not occur in isolation but may instead be closely linked to the accompanying inflammatory response [3, 15]. A clearer understanding of thyroid‐axis alterations and inflammation‐endocrine interactions may therefore help refine the interpretation of early hormonal changes and their potential relevance to longer‐term functional recovery after traumatic brain injury [3, 15, 16].

However, integrated clinical analyses examining temporal endocrine alterations, early inflammatory status, and longer‐term functional outcome within the same TBI cohort remain scarce [5, 11, 15, 17]. Therefore, the present study aimed to characterize temporal patterns of acute multi‐axis endocrine alterations in hospitalized patients with TBI and to explore their associations with 6‐month functional outcome. Secondary analyses examined associations with admission injury severity and day‐1 inflammatory markers. We further explored whether selected thyroid‐axis alterations and inflammatory markers were associated with unfavorable functional recovery.

## 2 Materials and Methods

### 2.1 Study Design and Participants

This single‐center prospective observational cohort study was conducted in the Department of Neurosurgery at the General Hospital of Ningxia Medical University. Consecutive hospitalized adults with traumatic brain injury were screened for eligibility between January 2024 and May 2025.

Patients were eligible if they met all of the following criteria: (1) age ≥ 18 years; (2) presentation within 24 h after head injury; (3) availability of post‐injury cranial computed tomography or magnetic resonance imaging. Patients were excluded if they had any of the following: (1) major extracranial injury requiring acute specialist treatment; (2) a documented history of hypothalamic–pituitary, thyroid, adrenal, or gonadal disease, or pre‐injury long‐term use of glucocorticoids, thyroid hormone, or other medications known to affect endocrine function; or (3) severe infection, immune‐mediated disease, or malignancy. For the final analysis, only patients with complete 6‐month Glasgow Outcome Scale‐Extended (GOS‐E) follow‐up data were included.

The study protocol was approved by the Ethics Committee of the General Hospital of Ningxia Medical University (Approval No. KYLL‐2024‐0014). Written informed consent was obtained from each patient or from a legally authorized representative before enrollment. The study was conducted in accordance with the Declaration of Helsinki.

### 2.2 Clinical Data Collection and Grouping

Demographic and clinical variables were collected, including age, sex, body mass index, blood pressure, blood glucose, serum sodium, associated injuries, admission Glasgow Coma Scale (GCS) score, neurosurgical intervention, neuroimaging findings, and major comorbidities. Neuroimaging comprised routine cranial CT or MRI for clinical TBI assessment and did not include dedicated pituitary MRI. Neurosurgical intervention was defined as craniotomy for evacuation of an intracranial hematoma or contusion, decompressive craniectomy with evacuation of an intracranial hematoma or contusion, surgery for skull fracture, or external ventricular drainage (EVD) placement alone during the index hospitalization. Each patient was classified according to the principal intervention. Concomitant EVD placement was not counted as a separate principal intervention. For the assessment of overall EVD use, however, both EVD placement alone and EVD performed concomitantly with another neurosurgical procedure were included. Associated injuries were defined as documented extracranial injuries that did not meet the exclusion criterion of major extracranial injury requiring acute specialist treatment. Patients were stratified into mild, moderate, and severe TBI groups according to admission GCS score, defined as 13–15, 9–12, and 3–8, respectively.

### 2.3 Hormone and Inflammatory Marker Assessment

Venous blood samples were prospectively collected on Day 1, 7, and 14 after injury. Inflammatory markers were measured on Day 1 only. Day 1 was selected to characterize the early post‐injury inflammatory response at the same standardized time point as the initial endocrine assessment, consistent with clinical evidence demonstrating detectable alterations in circulating IL‐6 and broader inflammatory profiles within 24 h after TBI [18, 19]. Serial inflammatory measurements were not included in the study protocol. The hormone panel included hypothalamic–pituitary–adrenal (HPA) axis markers, namely adrenocorticotropic hormone (ACTH) and serum cortisol measured at 08:00 and 16:00; thyroid‐axis markers, including triiodothyronine (T3), thyroxine (T4), free triiodothyronine (FT3), free thyroxine (FT4), and thyroid‐stimulating hormone (TSH); gonadal‐axis hormones, including luteinizing hormone (LH), follicle‐stimulating hormone (FSH), prolactin (PRL), testosterone, estradiol, and progesterone; and human growth hormone (hGH). Gonadal‐axis hormone analyses were restricted to male patients because menstrual cycle phase and menopausal status were not systematically recorded and may substantially affect gonadal hormone levels in female patients. Inflammatory markers included white blood cell count (WBC), neutrophil count (NEU), interleukin‐6 (IL‐6), and procalcitonin (PCT). These markers were selected to represent complementary components of the early systemic inflammatory response. Leukocyte and neutrophil counts were included as routinely available measures of the circulating cellular response after TBI [20]. IL‐6 is one of the most extensively studied acute inflammatory cytokines after TBI [18]. PCT has also been evaluated as a systemic inflammatory marker in relation to TBI severity and clinical outcome [21]. The panel was exploratory rather than comprehensive.

Blood for ACTH measurement was collected into prechilled ethylenediaminetetraacetic acid (EDTA) anticoagulant tubes and transported promptly for processing. Blood for cortisol, thyroid hormones, gonadal hormones, hGH, IL‐6, and PCT was collected into serum‐separation tubes without anticoagulant. Serum and plasma were separated by centrifugation, and all samples were analyzed on the same day of collection.

Hormone concentrations and immunoassay‐based inflammatory markers were measured in the hospital laboratory using the Atellica IM 1600 immunoassay system (Siemens Healthineers, Germany) in accordance with the manufacturer's instructions. WBC and NEU values were obtained from routine complete blood count testing performed by the hospital laboratory. Laboratory reference intervals were based on the standards provided by the hospital laboratory for the corresponding assay platform. Detailed reference ranges are provided in Supplementary Table S1. A schematic overview of the study design and assessment schedule is shown in Figure 1.

**FIGURE 1:**
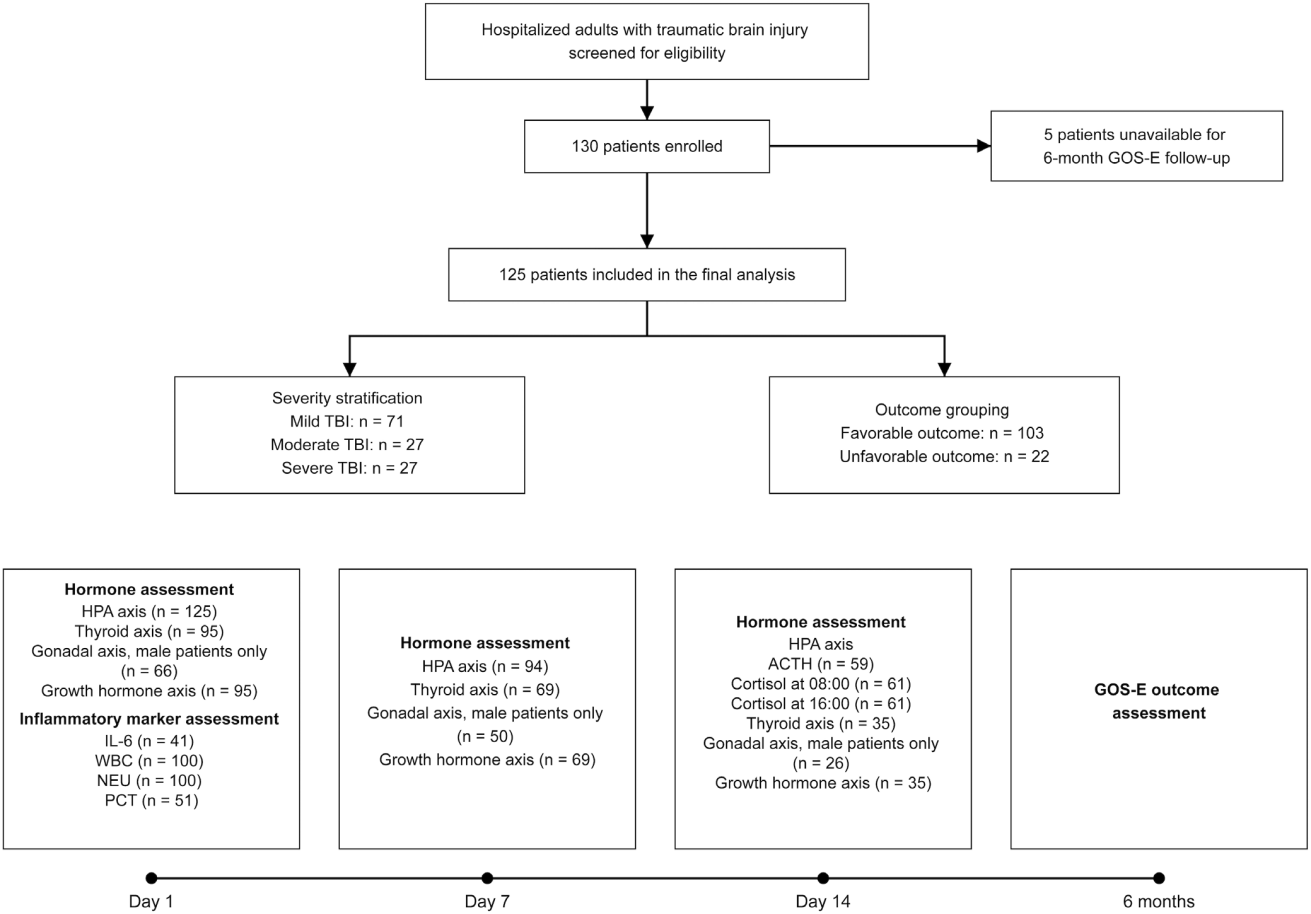
Study design and assessment schedule. Hospitalized adults with traumatic brain injury were screened and enrolled, and patients with complete 6‐month GOS‐E follow‐up data were included in the final analysis. Hormone assessments were performed on Day 1, 7, and 14 after injury. Inflammatory markers were measured on Day 1, and functional outcome was assessed at 6 months. Numbers in the assessment boxes indicate the number of patients with available measurements for each listed assessment at the corresponding time point. Gonadal‐axis hormone assessments were restricted to male patients.

### 2.4 Outcome Assessment

Functional outcome was prospectively assessed at 6 months after injury using the Glasgow Outcome Scale–Extended (GOS‐E) during outpatient follow‐up by trained neurosurgeons [22]. The GOS‐E was selected because it provides a standardized assessment of global functional status after TBI across eight categories ranging from death (score 1) to upper good recovery (score 8). Patients were classified into a favorable outcome group (GOS‐E scores 5–8) and an unfavorable outcome group (GOS‐E scores 1–4).

### 2.5 Statistical Analysis

Statistical analyses were performed using SPSS software, with Firth penalized logistic regression and effect‐size estimation conducted in R version 4.5.2 using the logistf (version 1.26.1) and effectsize (version 1.0.3) packages, respectively. Because most hormone and inflammatory variables were not normally distributed, continuous variables are presented as median (P25, P75) and categorical variables as number (percentage). For comparisons among mild, moderate, and severe TBI groups, continuous variables were analyzed using the Kruskal–Wallis test, followed by Bonferroni‐corrected pairwise Mann–Whitney *U* tests when the overall difference was significant. Categorical variables were compared using the chi‐squared test or Fisher's exact test, as appropriate.

To assess within‐patient changes in hormone levels across Day 1, 7, and 14, the Friedman test was used in patients with paired measurements available at all three time points for each hormone. When the overall difference was significant, post hoc pairwise comparisons were performed using the Wilcoxon signed‐rank test with Bonferroni correction. For comparisons between favorable and unfavorable outcome groups, continuous variables were analyzed using the Mann–Whitney *U* test, and categorical variables were compared using the chi‐squared test or Fisher's exact test, as appropriate.

Spearman's rank correlation analysis was used to evaluate the associations between day‐1 inflammatory markers and hormone levels measured on Day 1, 7, and 14. Correlation analyses involving sex‐hormone‐axis levels were restricted to male patients. Effect sizes were reported with 95% confidence intervals (CIs). Rank epsilon‐squared (*ε* ^2^), Cramér's *V* or phi, rank‐biserial *r*, Kendall's *W*, paired rank‐biserial *r*, and Spearman's *r* were used for Kruskal–Wallis, categorical, Mann–Whitney *U*, Friedman, Wilcoxon signed‐rank, and correlation analyses, respectively. Given the limited number of unfavorable outcome events, Firth penalized logistic regression was used to reduce small‐sample bias and improve the stability of the estimates. Separate models were fitted for IL‐6, WBC, NEU, PCT, day‐1 T3, and day‐1 FT3, with continuous admission GCS included in each model. Each model was based on complete cases. PCT was expressed per 0.1‐ng/mL increase, and adjusted odds ratios (ORs) with profile penalized‐likelihood 95% CIs were reported. All tests were two‐sided, and a *p* value < 0.05 was considered statistically significant. This manuscript was prepared in accordance with the Strengthening the Reporting of Observational Studies in Epidemiology (STROBE) reporting guideline.

## 3 Results

### 3.1 Baseline Characteristics According to TBI Severity

Among 130 enrolled patients, 125 with complete 6‐month GOS‐E assessment were included in the final analysis, including 71 with mild TBI, 27 with moderate TBI, and 27 with severe TBI. Baseline characteristics according to TBI severity are summarized in Table 1. No significant between‐group differences were observed in age, sex distribution, body mass index, systolic blood pressure, diastolic blood pressure, serum sodium level, or associated injuries. Height, body weight, and blood glucose differed significantly across the three severity groups. The proportion of patients undergoing neurosurgical intervention increased with injury severity, from 19.7% in mild TBI to 40.7% in moderate TBI and 59.3% in severe TBI. Among the 14 patients with mild TBI who underwent an intervention, seven underwent external ventricular drainage placement alone, six underwent surgery for skull fracture, and one underwent craniotomy for evacuation of an intracranial hematoma or contusion. In the moderate TBI group, nine patients underwent craniotomy for evacuation of an intracranial hematoma or contusion, one underwent surgery for skull fracture, and one underwent external ventricular drainage placement alone. In the severe TBI group, 10 patients underwent craniotomy for evacuation of an intracranial hematoma or contusion and six underwent decompressive craniectomy with evacuation of an intracranial hematoma or contusion. When both EVD placement alone and EVD performed concomitantly with another neurosurgical procedure were considered, EVD was performed in seven of 71 patients with mild TBI (9.9%), six of 27 patients with moderate TBI (22.2%), and nine of 27 patients with severe TBI (33.3%), for a total of 22 of 125 patients (17.6%).

**TABLE 1:** Baseline characteristics according to TBI severity.

Variable	Mild TBI (*n* = 71)	Moderate TBI (*n* = 27)	Severe TBI (*n* = 27)	*p*	Effect size (95% CI)
Age, years	53.0 (37.0, 59.0)	47.0 (29.0, 58.0)	45.0 (34.0, 57.0)	0.343	0.017 (0.001, 0.108)
Male sex, *n* (%)	46 (64.8%)	20 (74.1%)	21 (77.8%)	0.390	0.123 (0.000, 0.276)
Height, cm	169.0 (162.0, 175.0)	169.0 (160.0, 175.0)	173.0 (168.0, 178.0)	0.014	0.069 (0.014, 0.183)
Weight, kg	65.0 (56.0, 75.0)	65.0 (60.0, 73.0)	71.0 (63.0, 82.0)	0.020	0.063 (0.012, 0.178)
BMI, kg/m^2^	23.3 (21.3, 25.6)	23.8 (21.8, 25.0)	24.3 (22.3, 26.3)	0.417	0.014 (0.001, 0.096)
Systolic blood pressure, mmHg	131.0 (123.0, 142.0)	130.0 (122.0, 152.0)	136.0 (123.0, 146.0)	0.659	0.007 (0.001, 0.077)
Diastolic blood pressure, mmHg	78.0 (74.0, 86.0)	82.0 (69.0, 87.0)	82.0 (76.0, 90.0)	0.170	0.029 (0.003, 0.119)
Blood glucose, mmol/L	6.51 (5.87, 7.83)	7.12 (6.40, 10.02)	7.30 (6.39, 9.30)	0.028	0.057 (0.010, 0.175)
Serum sodium, mmol/L	139.5 (136.9, 142.2)	139.8 (137.6, 141.7)	140.5 (137.7, 142.0)	0.862	0.003 (0.001, 0.077)
Associated injury, *n* (%)	8 (11.3%)	3 (11.1%)	7 (25.9%)	0.199	0.172 (0.000, 0.331)
Neurosurgical intervention, *n* (%)	14 (19.7%)	11 (40.7%)	16 (59.3%)	< 0.001	0.345 (0.152, 0.511)

*Note:* Data are presented as median (P25, P75) or *n* (%). *p* values were calculated using the Kruskal–Wallis test for continuous variables and the *χ* ^2^ test or Fisher–Freeman–Halton exact test for categorical variables, as appropriate. Serum sodium values were available for 100 patients. Effect sizes are reported as rank epsilon‐squared (*ε* ^2^) for continuous variables and Cramér's *V* for categorical variables.

Abbreviations: CI, confidence interval; TBI, traumatic brain injury.

### 3.2 Temporal Changes in Hormone Levels After TBI

Descriptive hormone levels at each time point were summarized using available observations, whereas temporal changes across Day 1, 7, and 14 were assessed using paired data available at all three time points for each hormone. Temporal changes in hormone levels after TBI are summarized in Supplementary Table S2, and representative dynamic patterns are illustrated in Figure 2. Within the hypothalamic–pituitary–adrenal axis, ACTH did not change significantly across Day 1, 7, and 14 (Kendall's *W* = 0.004 [95% CI 0.001–0.076], *p* = 0.812). In contrast, serum cortisol at both 08:00 and 16:00 showed significant overall changes over time (Kendall's *W* = 0.205 [95% CI 0.085–0.388] and 0.239 [95% CI 0.126–0.408], respectively; both *p* < 0.001), with lower levels on Day 7 and 14 than on Day 1 after Bonferroni correction. Notably, although serum cortisol levels decreased over time, median cortisol at 08:00 remained above the laboratory reference range at all three time points. Within the thyroid axis, T3 showed a modest but significant temporal change (Kendall's *W* = 0.103 [95% CI 0.018–0.308], *p* = 0.045), FT3 increased significantly over time (Kendall's *W* = 0.168 [95% CI 0.043–0.404], *p* = 0.007), and TSH increased markedly from Day 1 to 7 and Day 14 (Kendall's *W* = 0.303 [95% CI 0.138–0.541], *p* < 0.001). No significant temporal changes were observed for T4 or FT4. Among male patients, testosterone increased significantly over time (Kendall's *W* = 0.306 [95% CI 0.101–0.620], *p* = 0.001), whereas progesterone decreased significantly from Day 1 to 7 and Day 14 (Kendall's *W* = 0.666 [95% CI 0.513–0.820], *p* < 0.001). LH, FSH, prolactin, estradiol, and hGH did not show significant temporal changes.

**FIGURE 2:**
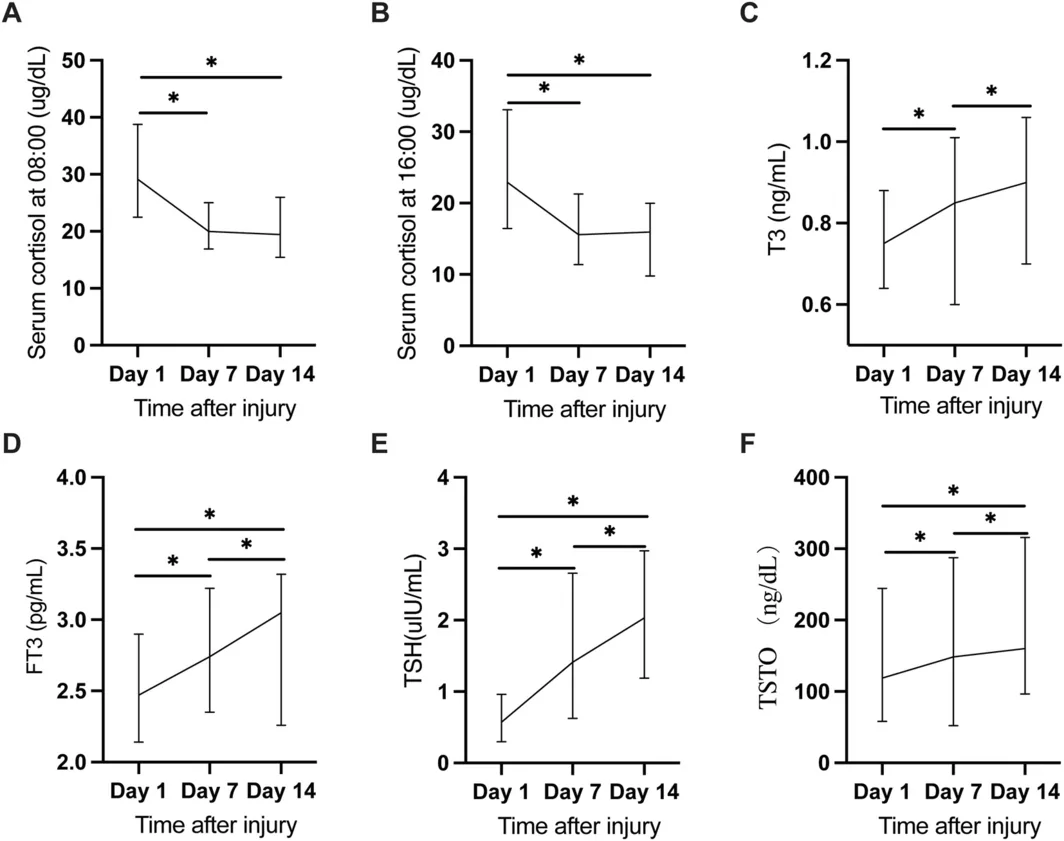
Dynamic changes in selected serum hormone levels after traumatic brain injury. Panels show changes in (A) serum cortisol at 08:00, (B) serum cortisol at 16:00, (C) T3, (D) FT3, (E) TSH, and (F) TSTO on Days 1, 7, and 14 after traumatic brain injury. Data are presented as median (P25, P75). *p < 0.017 after Bonferroni correction. Corresponding effect sizes and 95% CIs are provided in Supplementary Table S2. TSTO was analyzed in male patients only. FT3, free triiodothyronine; T3, triiodothyronine; TSH, thyroid‐stimulating hormone; TSTO, testosterone.

### 3.3 Hormone Levels According to TBI Severity

Comparisons of HPA‐axis hormones, thyroid‐axis hormones, and hGH across severity groups are presented in Table 2. On Day 1, serum cortisol at 16:00 differed significantly among the mild, moderate, and severe TBI groups (rank epsilon‐squared [*ε* ^2^] = 0.090 [95% CI 0.022–0.219], *p* = 0.004), with post hoc analysis showing higher levels in moderate TBI than in mild TBI (rank‐biserial *r* = −0.391 [95% CI −0.585 to −0.154], Bonferroni‐corrected *p* = 0.009). By contrast, serum cortisol at 08:00 did not reach statistical significance. On Day 1, median cortisol levels at both 08:00 and 16:00 exceeded the laboratory reference range in all three severity groups. ACTH did not differ significantly across GCS‐defined severity groups at any time point. Within the thyroid axis, day‐1 FT3 differed significantly across severity groups (rank epsilon‐squared [*ε* ^2^] = 0.110 [95% CI 0.020–0.291], *p* = 0.006), with lower FT3 levels in severe TBI than in both mild and moderate TBI. No significant between‐group differences were observed for day‐1 T3, T4, FT4, or TSH.

**TABLE 2:** Comparison of HPA‐axis, thyroid‐axis, and hGH levels at different time points according to TBI severity.

Time point	Variable	Mild TBI	Moderate TBI	Severe TBI	*p*	Significant post hoc comparison	Effect size (95% CI)
Day 1	ACTH	26.76 (14.63, 49.64)	32.53 (21.10, 43.90)	39.76 (13.46, 74.31)	0.562	—	*ε* ^2^ = 0.009 (0.001, 0.091)
Day 1	Cortisol at 08:00	28.62 (19.55, 35.90)	32.94 (23.35, 42.16)	30.57 (23.98, 39.42)	0.082	—	*ε* ^2^ = 0.040 (0.004, 0.145)
Day 1	Cortisol at 16:00	19.83 (13.36, 25.41)	27.39 (21.87, 37.13)	23.57 (18.43, 36.52)	0.004	Mild vs. moderate, *p* = 0.009	*ε* ^2^ = 0.090 (0.022, 0.219) Mild vs. moderate: *r* = −0.391 (−0.585, −0.154)
Day 1	T3	0.77 (0.66, 0.87)	0.79 (0.71, 1.08)	0.65 (0.48, 0.87)	0.084	—	*ε* ^2^ = 0.053 (0.005, 0.205)
Day 1	T4	6.90 (6.10, 8.10)	6.50 (4.90, 9.50)	6.20 (4.70, 8.50)	0.648	—	*ε* ^2^ = 0.009 (0.001, 0.122)
Day 1	FT3	2.47 (2.25, 2.78)	2.83 (2.28, 3.17)	2.14 (1.68, 2.51)	0.006	Mild vs. severe, *p* = 0.037; Moderate vs. severe, *p* = 0.021	*ε* ^2^ = 0.110 (0.020, 0.291) Mild vs. severe: *r* = 0.401 (0.111, 0.628) Moderate vs. severe: *r* = 0.526 (0.199, 0.748)
Day 1	FT4	1.13 (1.01, 1.30)	1.11 (1.02, 1.39)	1.11 (0.89, 1.29)	0.662	—	*ε* ^2^ = 0.009 (0.001, 0.109)
Day 1	TSH	0.607 (0.314, 1.028)	0.536 (0.276, 0.935)	0.433 (0.200, 1.150)	0.533	—	*ε* ^2^ = 0.013 (0.001, 0.115)
Day 1	hGH	0.612 (0.291, 1.900)	0.908 (0.513, 1.840)	0.574 (0.316, 1.122)	0.214	—	*ε* ^2^ = 0.033 (0.003, 0.134)
Day 7	ACTH	38.48 (25.39, 50.63)	32.19 (26.66, 49.09)	28.16 (15.33, 58.02)	0.531	—	*ε* ^2^ = 0.014 (0.001, 0.124)
Day 7	Cortisol at 08:00	18.90 (13.98, 24.39)	22.16 (19.14, 27.96)	19.94 (15.05, 21.35)	0.016	Mild vs. moderate, *p* = 0.021	*ε* ^2^ = 0.089 (0.019, 0.235) Mild vs. moderate: *r* = −0.396 (−0.609, −0.130)
Day 7	Cortisol at 16:00	14.27 (9.19, 20.40)	17.30 (12.65, 21.04)	18.34 (11.99, 26.19)	0.092	—	*ε* ^2^ = 0.051 (0.006, 0.185)
Day 7	T3	0.90 (0.71, 1.06)	0.84 (0.61, 1.00)	0.68 (0.50, 0.91)	0.048	Mild vs. severe, *p* = 0.045	*ε* ^2^ = 0.089 (0.011, 0.272) Mild vs. severe: *r* = 0.417 (0.107, 0.653)
Day 7	T4	8.50 (7.13, 9.90)	7.20 (5.23, 9.43)	6.30 (3.70, 8.65)	0.006	Mild vs. severe, *p* = 0.011	*ε* ^2^ = 0.150 (0.034, 0.377) Mild vs. severe: *r* = 0.497 (0.206, 0.707)
Day 7	FT3	2.83 (2.49, 3.35)	2.89 (2.31, 3.22)	2.42 (1.73, 2.80)	0.021	Mild vs. severe, *p* = 0.025	*ε* ^2^ = 0.113 (0.017, 0.314) Mild vs. severe: *r* = 0.453 (0.151, 0.677)
Day 7	FT4	1.24 (1.15, 1.37)	1.21 (1.07, 1.41)	1.01 (0.71, 1.26)	0.009	Mild vs. severe, *p* = 0.007	*ε* ^2^ = 0.139 (0.028, 0.358) Mild vs. severe: *r* = 0.523 (0.239, 0.724)
Day 7	TSH	1.996 (1.146, 3.670)	1.704 (0.612, 2.613)	0.437 (0.219, 1.286)	0.002	Mild vs. severe, *p* = 0.001; Moderate vs. severe, *p* = 0.039	*ε* ^2^ = 0.191 (0.064, 0.391) Mild vs. severe: *r* = 0.608 (0.353, 0.779) Moderate vs. severe: *r* = 0.507 (0.158, 0.744)
Day 7	hGH	0.682 (0.343, 1.295)	0.915 (0.531, 2.263)	0.958 (0.534, 1.825)	0.274	—	*ε* ^2^ = 0.038 (0.002, 0.191)
Day 14	ACTH	27.68 (19.27, 44.17)	36.18 (17.74, 61.71)	33.85 (22.49, 52.21)	0.602	—	*ε* ^2^ = 0.018 (0.002, 0.174)
Day 14	Cortisol at 08:00	19.84 (16.80, 27.97)	24.23 (18.83, 26.84)	17.44 (11.92, 20.28)	0.040	Moderate vs. severe, *p* = 0.046	*ε* ^2^ = 0.108 (0.018, 0.333) Moderate vs. severe: *r* = 0.449 (0.119, 0.689)
Day 14	Cortisol at 16:00	17.37 (13.32, 20.99)	17.47 (9.14, 21.16)	11.15 (9.26, 17.37)	0.135	—	*ε* ^2^ = 0.067 (0.007, 0.259)
Day 14	T3	0.89 (0.82, 1.01)	0.87 (0.71, 1.01)	0.92 (0.53, 1.11)	0.946	—	*ε* ^2^ = 0.003 (0.002, 0.205)
Day 14	T4	9.70 (6.45, 11.35)	6.90 (5.70, 8.85)	7.25 (4.85, 9.20)	0.106	—	*ε* ^2^ = 0.132 (0.015, 0.438)
Day 14	FT3	3.02 (2.64, 3.43)	3.10 (2.38, 3.28)	2.88 (2.17, 3.35)	0.760	—	*ε* ^2^ = 0.016 (0.002, 0.237)
Day 14	FT4	1.30 (1.15, 1.48)	1.12 (1.00, 1.26)	1.09 (0.90, 1.41)	0.196	—	*ε* ^2^ = 0.096 (0.012, 0.373)
Day 14	TSH	2.184 (1.142, 3.323)	2.070 (1.481, 3.598)	1.987 (1.090, 2.847)	0.675	—	*ε* ^2^ = 0.023 (0.002, 0.269)
Day 14	hGH	1.325 (0.257, 2.628)	1.040 (0.412, 2.300)	1.035 (0.434, 1.778)	0.900	—	*ε* ^2^ = 0.006 (0.002, 0.238)

*Note:* Data are presented as median (P25, P75). Measurement units are provided in Supplementary Table S1. *p* values were calculated using the Kruskal–Wallis test. Post hoc pairwise comparisons were performed using the Mann–Whitney *U* test with Bonferroni correction when the Kruskal–Wallis test was significant. Effect sizes are reported as rank epsilon‐squared (*ε* ^2^) for omnibus comparisons and rank‐biserial *r* for significant post hoc pairwise comparisons.

Abbreviations: ACTH, adrenocorticotropic hormone; CI, confidence interval; FT3, free triiodothyronine; FT4, free thyroxine; hGH, human growth hormone; HPA, hypothalamic–pituitary–adrenal; T3, triiodothyronine; T4, thyroxine; TBI, traumatic brain injury; TSH, thyroid‐stimulating hormone.

On Day 7, several thyroid‐axis markers differed significantly across severity groups. T3, T4, FT3, FT4, and TSH were lower in severe TBI than in mild TBI on post hoc testing, and TSH was also lower in severe TBI than in moderate TBI. Serum cortisol at 08:00 also differed significantly across groups on Day 7, with higher levels in moderate TBI than in mild TBI. On Day 14, serum cortisol at 08:00 differed significantly across severity groups, mainly because of lower levels in severe TBI than in moderate TBI. No significant between‐group differences were observed for hGH at any time point.

Gonadal‐axis analyses were restricted to male patients, and the results are summarized in Table 3. No significant between‐group differences were observed for male gonadal hormones on Day 1. On Day 7, FSH, PRL, and testosterone differed significantly across severity groups. Post hoc analysis showed lower FSH and testosterone levels in severe TBI than in mild TBI, and lower PRL levels in severe TBI than in both mild and moderate TBI. No significant between‐group differences were observed for estradiol or progesterone on Day 7. On Day 14, no male gonadal hormone showed a statistically significant difference across severity groups.

**TABLE 3:** Comparison of male gonadal hormone levels at different time points according to TBI severity.

Time point	Variable	Mild TBI (*n* = 46)	Moderate TBI (*n* = 20)	Severe TBI (*n* = 21)	*p*	Significant post hoc comparison	Effect size (95% CI)
Day 1	LH	3.79 (2.73, 5.04)	5.16 (3.67, 7.44)	3.20 (2.42, 4.75)	0.091	—	*ε* ^2^ = 0.074 (0.008, 0.249)
Day 1	FSH	5.54 (4.38, 8.02)	7.22 (3.85, 8.66)	4.10 (2.00, 8.96)	0.276	—	*ε* ^2^ = 0.040 (0.003, 0.234)
Day 1	PRL	9.40 (6.73, 12.09)	8.69 (7.35, 12.28)	8.71 (6.77, 10.57)	0.836	—	*ε* ^2^ = 0.005 (0.001, 0.119)
Day 1	TSTO	162.43 (67.94, 245.82)	106.51 (43.91, 213.59)	55.75 (24.46, 217.19)	0.125	—	*ε* ^2^ = 0.064 (0.006, 0.261)
Day 1	E2	29.45 (22.78, 36.65)	30.73 (25.74, 39.93)	26.32 (21.66, 33.37)	0.539	—	*ε* ^2^ = 0.019 (0.002, 0.159)
Day 1	PRGE	0.465 (0.248, 0.748)	0.755 (0.428, 1.383)	0.395 (0.243, 0.670)	0.121	—	*ε* ^2^ = 0.065 (0.007, 0.246)
Day 7	LH	4.46 (3.22, 6.42)	4.85 (2.84, 6.78)	2.65 (1.24, 5.22)	0.093	—	*ε* ^2^ = 0.097 (0.011, 0.337)
Day 7	FSH	6.30 (4.41, 9.55)	5.56 (3.20, 8.66)	2.79 (1.94, 5.60)	0.039	Mild vs. severe, *p* = 0.031	*ε* ^2^ = 0.132 (0.018, 0.381) Mild vs. severe: *r* = 0.531 (0.184, 0.761)
Day 7	PRL	11.67 (9.40, 14.70)	13.58 (11.48, 14.61)	8.08 (2.49, 12.40)	0.014	Mild vs. severe, *p* = 0.043; Moderate vs. severe, *p* = 0.035	*ε* ^2^ = 0.174 (0.041, 0.422) Mild vs. severe: *r* = 0.507 (0.152, 0.746) Moderate vs. severe: *r* = 0.583 (0.211, 0.808)
Day 7	TSTO	261.31 (125.68, 424.40)	116.98 (63.89, 191.81)	35.50 (23.08, 72.36)	< 0.001	Mild vs. severe, *p* = 0.003	*ε* ^2^ = 0.282 (0.081, 0.561) Mild vs. severe: *r* = 0.674 (0.390, 0.841)
Day 7	E2	30.04 (21.38, 38.38)	29.85 (25.34, 37.06)	25.88 (16.70, 29.20)	0.277	—	*ε* ^2^ = 0.052 (0.004, 0.241)
Day 7	PRGE	0.295 (0.110, 0.508)	0.320 (0.110, 0.380)	0.110 (0.110, 0.385)	0.555	—	*ε* ^2^ = 0.024 (0.002, 0.206)
Day 14	LH	3.35 (2.14, 6.15)	4.53 (3.50, 6.08)	3.54 (2.66, 7.38)	0.612	—	*ε* ^2^ = 0.039 (0.004, 0.360)
Day 14	FSH	8.59 (5.92, 12.07)	5.67 (3.70, 7.31)	4.39 (3.46, 6.59)	0.135	—	*ε* ^2^ = 0.160 (0.037, 0.472)
Day 14	PRL	20.05 (12.96, 26.56)	11.35 (9.87, 13.57)	12.17 (6.10, 14.33)	0.107	—	*ε* ^2^ = 0.179 (0.018, 0.534)
Day 14	TSTO	291.73 (154.84, 540.60)	226.20 (120.46, 366.68)	121.15 (35.79, 254.24)	0.051	—	*ε* ^2^ = 0.237 (0.046, 0.589)
Day 14	E2	26.41 (15.12, 44.37)	32.21 (24.49, 45.35)	24.30 (15.02, 34.27)	0.327	—	*ε* ^2^ = 0.089 (0.009, 0.451)
Day 14	PRGE	0.430 (0.110, 0.515)	0.290 (0.110, 0.440)	0.110 (0.110, 0.368)	0.364	—	*ε* ^2^ = 0.081 (0.007, 0.476)

*Note:* Data are presented as median (P25, P75). *p* values were calculated using the Kruskal–Wallis test; post hoc pairwise comparisons were performed using the Mann–Whitney *U* test with Bonferroni correction. Gonadal‐axis hormones were analyzed in male patients only. The numbers in the column headings indicate the total numbers of male patients in each TBI severity category. Effect sizes are reported as rank epsilon‐squared (*ε* ^2^) for omnibus comparisons and rank‐biserial *r* for significant post hoc pairwise comparisons.

Abbreviations: CI, confidence interval; E2, estradiol; FSH, follicle‐stimulating hormone; LH, luteinizing hormone; PRGE, progesterone; PRL, prolactin; TBI, traumatic brain injury; TSTO, testosterone.

### 3.4 Day‐1 Inflammatory Markers and Their Correlations With Hormone Levels

Comparisons of day‐1 inflammatory markers across GCS‐defined severity groups are shown in Table 4. IL‐6, WBC, NEU, and PCT all differed significantly among the mild, moderate, and severe TBI groups. Post hoc analysis showed higher IL‐6, WBC, and NEU levels in severe TBI than in mild TBI. PCT levels were significantly higher in moderate than in mild TBI and in severe than in both mild and moderate TBI.

**TABLE 4:** Inflammatory marker levels on Day 1 according to TBI severity.

Variable	Mild TBI	Moderate TBI	Severe TBI	*p*	Significant post hoc comparison	Effect size (95% CI)
IL‐6	13.60 (9.82, 29.80)	36.90 (18.10, 63.90)	72.50 (34.80, 146.00)	0.002	Mild vs. severe, *p* = 0.004	*ε* ^2^ = 0.315 (0.101, 0.627) Mild vs. severe: *r* = −0.688 (−0.853, −0.397)
WBC	11.52 (9.31, 15.15)	12.85 (8.04, 17.05)	14.33 (11.75, 20.64)	0.023	Mild vs. severe, *p* = 0.014	*ε* ^2^ = 0.076 (0.016, 0.206) Mild vs. severe: *r* = −0.430 (−0.640, −0.159)
NEU	9.79 (7.36, 12.56)	9.73 (6.61, 15.08)	12.28 (9.44, 17.80)	0.048	Mild vs. severe, *p* = 0.033	*ε* ^2^ = 0.062 (0.012, 0.189) Mild vs. severe: *r* = −0.388 (−0.609, −0.110)
PCT	0.053 (0.029, 0.075)	0.106 (0.050, 0.148)	0.230 (0.120, 0.428)	< 0.001	Mild vs. moderate, *p* = 0.009; Mild vs. severe, *p* < 0.001; Moderate vs. severe, *p* = 0.028	*ε* ^2^ = 0.508 (0.303, 0.719) Mild vs. moderate: *r* = −0.631 (−0.821, −0.316) Mild vs. severe: *r* = −0.860 (−0.930, −0.728) Moderate vs. severe: *r* = −0.569 (−0.791, −0.215)

*Note:* Data are presented as median (P25, P75). *p* values were calculated using the Kruskal–Wallis test. Post hoc pairwise comparisons were performed using the Mann–Whitney *U* test with Bonferroni correction. Effect sizes are reported as rank epsilon‐squared (*ε* ^2^) for omnibus comparisons and rank‐biserial *r* for significant post hoc pairwise comparisons.

Abbreviations: CI, confidence interval; IL‐6, interleukin‐6; NEU, neutrophil count; PCT, procalcitonin; TBI, traumatic brain injury; WBC, white blood cell count.

Correlations between day‐1 inflammatory markers and core hormone levels are summarized in Supplementary Table S3. In the same‐day analysis, IL‐6 was positively correlated with day‐1 serum cortisol at 16:00 (*r* = 0.498 [95% CI 0.207–0.708], *p* < 0.001), whereas WBC and NEU were negatively correlated with day‐1 T4 (*r* = −0.311 [95% CI −0.486 to −0.112], *p* = 0.002; *r* = −0.337 [95% CI −0.509 to −0.140], *p* < 0.001, respectively). PCT was negatively correlated with day‐1 FT3 (*r* = −0.353 [95% CI −0.587 to −0.064], *p* = 0.015). These same‐day correlations are illustrated in Figure 3. Day‐7 serum cortisol at 16:00 was positively correlated with day‐1 IL‐6, whereas day‐7 T4 and TSH were negatively correlated with day‐1 IL‐6. Day‐1 WBC and day‐1 NEU were also negatively correlated with several day‐7 thyroid‐axis markers, most consistently T3, T4, and FT4. Day‐1 WBC and day‐1 NEU remained negatively correlated with selected day‐14 thyroid‐axis markers, especially T4.

**FIGURE 3:**
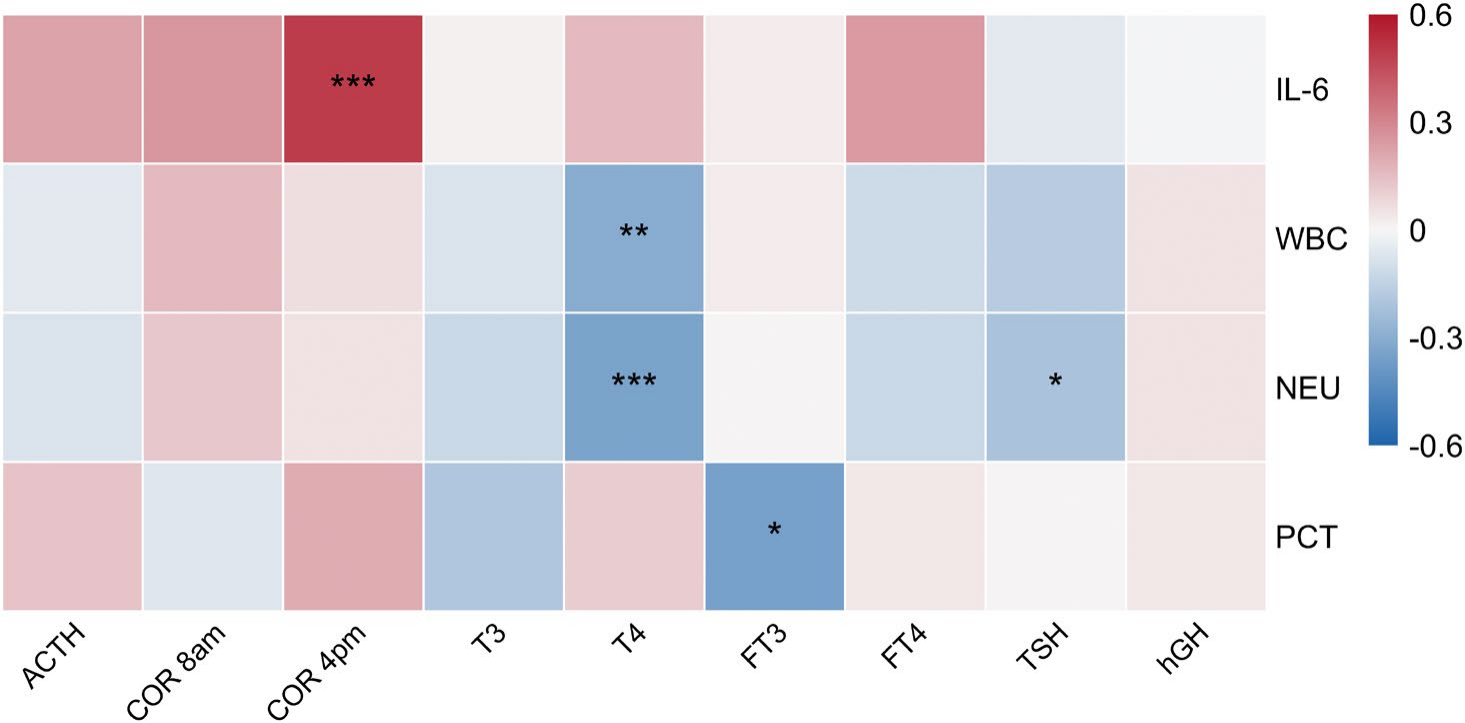
Correlations between inflammatory markers and hormone levels on Day 1 after traumatic brain injury. Heatmap showing Spearman correlations between day‐1 inflammatory markers and day‐1 core hormone levels after traumatic brain injury. *p < 0.05, **p < 0.01, ***p < 0.001. Corresponding Spearman correlation coefficients and their 95% CIs are provided in Supplementary Table S3.

Correlations between day‐1 inflammatory markers and sex‐hormone‐axis levels in male patients are provided in Supplementary Table S4. In male patients, day‐1 WBC and day‐1 NEU were negatively correlated with testosterone measured at multiple time points, including Day 1, 7, and 14.

### 3.5 Clinical and Hormonal Comparisons According to 6‐Month Outcome After TBI

Patients were classified into favorable (*n* = 103) and unfavorable (*n* = 22) outcome groups according to 6‐month GOS‐E outcome after TBI. Comparisons of clinical characteristics, inflammatory markers, and core hormone levels between the two outcome groups are shown in Table 5. Patients with unfavorable outcome had markedly lower admission GCS scores than those with favorable outcome (rank‐biserial *r* = 0.966, 95% CI 0.942–0.980; *p* < 0.001), whereas no significant between‐group differences were observed in age or sex distribution. The unfavorable outcome group included two patients who died by 6 months (GOS‐E score 1), both of whom had severe TBI based on admission GCS. Day‐1 inflammatory markers were higher in the unfavorable outcome group, including IL‐6 (rank‐biserial *r* = −0.482, 95% CI −0.730 to −0.121; *p* = 0.019), PCT (rank‐biserial *r* = −0.652, 95% CI −0.809 to −0.408; *p* < 0.001), and NEU (rank‐biserial *r* = −0.306, 95% CI −0.550 to −0.014; *p* = 0.047), whereas WBC did not reach statistical significance.

**TABLE 5:** Comparison of clinical characteristics, inflammatory markers, and core hormone levels between patients with favorable and unfavorable 6‐month outcomes.

Variable	Favorable outcome (*n* = 103)	Unfavorable outcome (*n* = 22)	*p*	Effect size (95% CI)
Clinical characteristics				
Age, years	52.00 (35.00, 59.00)	47.00 (34.75, 62.25)	0.899	−0.017 (−0.277, 0.245)
Male sex, *n* (%)	70 (68.0%)	17 (77.3%)	0.389	0.077 (0.000, 0.252)
GCS	15.00 (12.00, 15.00)	5.00 (3.00, 5.25)	< 0.001	0.966 (0.942, 0.980)
Day 1 inflammatory markers				
IL‐6	18.50 (11.50, 54.38)	72.50 (34.60, 137.00)	0.019	−0.482 (−0.730, −0.121)
WBC	11.57 (9.49, 16.49)	14.14 (10.88, 19.59)	0.070	−0.280 (−0.530, 0.015)
NEU	9.80 (7.36, 13.48)	12.28 (9.13, 17.21)	0.047	−0.306 (−0.550, −0.014)
PCT	0.073 (0.032, 0.126)	0.215 (0.113, 0.523)	< 0.001	−0.652 (−0.809, −0.408)
Day 1 core hormone levels				
Day 1 ACTH	29.37 (15.53, 49.64)	36.15 (12.05, 71.03)	0.758	−0.042 (−0.299, 0.221)
Day 1 cortisol at 08:00	29.08 (22.48, 38.31)	30.79 (22.88, 41.27)	0.383	−0.119 (−0.368, 0.146)
Day 1 cortisol at 16:00	22.78 (15.07, 30.01)	23.32 (18.27, 36.52)	0.310	−0.138 (−0.385, 0.127)
Day 1 T3	0.775 (0.668, 0.883)	0.640 (0.435, 0.870)	0.013	0.385 (0.102, 0.611)
Day 1 T4	6.90 (6.00, 8.15)	6.20 (4.55, 8.50)	0.163	0.216 (−0.084, 0.481)
Day 1 FT3	2.545 (2.260, 2.910)	2.130 (1.580, 2.390)	0.001	0.501 (0.241, 0.694)
Day 1 FT4	1.135 (1.020, 1.310)	1.060 (0.885, 1.240)	0.146	0.225 (−0.075, 0.488)
Day 1 TSH	0.582 (0.307, 1.061)	0.433 (0.200, 0.889)	0.351	0.145 (−0.157, 0.422)
Day 1 hGH	0.628 (0.421, 1.855)	0.570 (0.312, 0.834)	0.152	0.222 (−0.078, 0.486)
Day 7 core hormone levels				
Day 7 ACTH	35.45 (24.94, 50.16)	32.62 (12.53, 55.35)	0.577	0.080 (−0.199, 0.347)
Day 7 cortisol at 08:00	20.27 (17.09, 25.52)	19.14 (15.05, 21.26)	0.282	0.155 (−0.125, 0.412)
Day 7 cortisol at 16:00	15.52 (11.44, 21.02)	17.29 (10.80, 26.88)	0.369	−0.129 (−0.390, 0.151)
Day 7 T3	0.880 (0.690, 1.050)	0.655 (0.455, 0.913)	0.016	0.401 (0.099, 0.635)
Day 7 T4	8.00 (6.70, 9.65)	5.85 (3.75, 8.98)	0.020	0.386 (0.081, 0.624)
Day 7 FT3	2.840 (2.435, 3.235)	2.445 (1.850, 2.833)	0.020	0.387 (0.083, 0.625)
Day 7 FT4	1.220 (1.100, 1.370)	1.015 (0.743, 1.313)	0.015	0.404 (0.104, 0.638)
Day 7 TSH	1.928 (0.867, 3.534)	0.542 (0.201, 1.346)	< 0.001	0.550 (0.285, 0.737)
Day 7 hGH	0.856 (0.368, 1.310)	0.917 (0.576, 1.923)	0.374	−0.147 (−0.441, 0.175)
Day 14 core hormone levels				
Day 14 ACTH	30.09 (18.36, 45.12)	34.85 (24.15, 62.12)	0.343	−0.154 (−0.441, 0.161)
Day 14 cortisol at 08:00	22.22 (16.96, 27.42)	17.11 (11.46, 20.34)	0.025	0.360 (0.061, 0.599)
Day 14 cortisol at 16:00	17.31 (11.98, 20.64)	10.57 (6.95, 17.41)	0.036	0.337 (0.035, 0.582)
Day 14 T3	0.870 (0.760, 1.020)	0.915 (0.478, 1.130)	1.000	0.000 (−0.376, 0.376)
Day 14 T4	7.50 (6.00, 10.05)	7.25 (4.63, 9.25)	0.372	0.180 (−0.211, 0.521)
Day 14 FT3	3.050 (2.450, 3.340)	2.880 (2.093, 3.295)	0.429	0.160 (−0.231, 0.506)
Day 14 FT4	1.230 (1.050, 1.345)	1.085 (0.940, 1.435)	0.296	0.211 (−0.180, 0.544)
Day 14 TSH	2.432 (1.481, 3.434)	1.726 (1.017, 2.758)	0.121	0.313 (−0.072, 0.617)
Day 14 hGH	1.040 (0.271, 2.715)	1.035 (0.498, 1.593)	0.686	0.082 (−0.304, 0.444)

*Note:* Data are presented as median (P25, P75) or *n* (%). Comparisons were based on available samples for each variable. *p* values were calculated using the Mann–Whitney *U* test for continuous variables and the *χ* ^2^ test for categorical variables. Effect sizes are reported as rank‐biserial *r* for continuous variables and phi for male sex, with 95% CIs. Positive rank‐biserial *r* values indicate higher values in the favorable outcome group.

Abbreviations: ACTH, adrenocorticotropic hormone; CI, confidence interval; FT3, free triiodothyronine; FT4, free thyroxine; GCS, Glasgow Coma Scale; hGH, human growth hormone; IL‐6, interleukin‐6; NEU, neutrophil count; PCT, procalcitonin; T3, triiodothyronine; T4, thyroxine; TSH, thyroid‐stimulating hormone; WBC, white blood cell count.

With respect to core hormone levels, patients with unfavorable outcome had significantly lower day‐1 T3 (rank‐biserial *r* = 0.385, 95% CI 0.102–0.611; *p* = 0.013) and FT3 (rank‐biserial *r* = 0.501, 95% CI 0.241–0.694; *p* = 0.001) levels than those with favorable outcome. No significant between‐group differences were observed for day‐1 ACTH, serum cortisol at 08:00, serum cortisol at 16:00, T4, FT4, TSH, or hGH. At later time points, the unfavorable outcome group had lower day‐7 T3, T4, FT3, FT4, and TSH levels, and lower day‐14 serum cortisol levels at both 08:00 and 16:00. These comparisons were based on available samples for each variable and should therefore be interpreted cautiously.

Sex‐hormone‐axis levels were compared between favorable and unfavorable outcome groups among male patients, as shown in Supplementary Table S5. Day‐7 testosterone was significantly lower in the unfavorable outcome group than in the favorable outcome group (rank‐biserial *r* = 0.441, 95% CI 0.083–0.698; *p* = 0.027). No significant between‐group differences were observed for the other sex‐hormone‐axis markers at Day 1, 7, or 14.

### 3.6 GCS‐Adjusted Firth Penalized Logistic Regression Analyses of Factors Associated With Unfavorable 6‐Month Functional Outcome

Separate Firth penalized logistic regression models were fitted for IL‐6, WBC, NEU, PCT, day‐1 T3, and day‐1 FT3, with admission GCS included as a continuous covariate in each model. None of the six biomarkers were significantly associated with unfavorable 6‐month functional outcome (Table 6).

**TABLE 6:** Admission GCS‐adjusted Firth penalized logistic regression analyses of associations between biomarkers and unfavorable 6‐month functional outcome.

Variable	*n* (unfavorable events)	GCS‐adjusted OR (95% CI)	*p*
IL‐6	41 (11)	0.994 (0.975, 1.011)	0.473
WBC	100 (17)	0.892 (0.716, 1.059)	0.192
NEU	100 (17)	0.946 (0.769, 1.149)	0.577
PCT, per 0.1 ng/mL increase	51 (16)	0.987 (0.924, 1.115)	0.721
Day‐1 T3	95 (17)	0.029 (< 0.001, 9.568)	0.242
Day‐1 FT3	95 (17)	0.158 (< 0.001, 1.907)	0.192

*Note:* Each row represents a separate Firth penalized logistic regression model including continuous admission GCS score and the listed biomarker. The dependent variable was unfavorable 6‐month outcome (GOS‐E scores 1–4). Analyses were based on complete cases for each model. ORs are reported per 1‐unit increase except for PCT, which is reported per 0.1‐ng/mL increase. The 95% CIs were estimated using profile penalized likelihood.

Abbreviations: CI, confidence interval; FT3, free triiodothyronine; GCS, Glasgow Coma Scale; GOS‐E, Glasgow Outcome Scale‐Extended; IL‐6, interleukin‐6; NEU, neutrophil count; OR, odds ratio; PCT, procalcitonin; T3, triiodothyronine; WBC, white blood cell count.

## 4 Discussion

The main findings were that acute endocrine changes were heterogeneous and time‐dependent, with prominent changes involving cortisol, thyroid‐axis markers, and selected male gonadal hormones. Endocrine and inflammatory differences were also observed across GCS‐defined severity groups, particularly involving thyroid‐axis markers. Compared with patients with favorable outcome, those with unfavorable 6‐month functional outcome had higher day‐1 IL‐6 and PCT levels, higher NEU counts, and lower day‐1 T3 and FT3 levels. However, after adjustment for admission GCS in separate Firth penalized logistic regression models, IL‐6, WBC, NEU, PCT, day‐1 T3, and day‐1 FT3 were not significantly associated with unfavorable outcome. Previous work has described dynamic post‐traumatic endocrine changes across TBI severity groups [23]. The present study extends this literature by integrating serial multi‐axis endocrine measurements on Day 1, 7, and 14 with day‐1 inflammatory markers, GCS‐defined injury severity, and a prespecified 6‐month functional outcome assessment within the same prospective cohort. This integrated design allowed us to characterize endocrine trajectories during the first two post‐injury weeks, examine their relationships with early systemic inflammation and injury severity, and assess whether selected day‐1 biomarkers remained associated with 6‐month outcome after adjustment for admission GCS.

On Day 1, serum cortisol at 16:00 differed significantly across GCS‐defined severity groups, whereas FT3 was lower in severe TBI than in mild and moderate TBI. The cortisol findings should not be interpreted as a simple linear relationship between hormone level and injury severity, because acute cortisol concentrations after TBI are likely influenced not only by injury burden itself but also by activation of the hypothalamic–pituitary–adrenal axis, sympathetic stress responses, surgery, pain, hemodynamic instability, concomitant extracerebral trauma, and altered cortisol metabolism during critical illness [6, 23, 24, 25, 26, 27]. This interpretation is broadly consistent with clinical studies showing that cortisol levels are typically elevated early after severe TBI and tend to decline during recovery, while also varying according to disease severity, progression, and subsequent outcome [23, 24, 25, 26]. The thyroid‐axis findings showed a more consistent relationship with injury severity during the first post‐injury week. FT3 was lower in severe TBI than in mild and moderate TBI on Day 1, and all measured thyroid‐axis markers were lower in severe TBI than in mild TBI on Day 7. A previous study of severe TBI similarly reported reductions in T3 and T4 and associations between lower levels and greater injury severity [28]. More recently, Zhang et al. found that T3, FT3, and FT4 tended to decrease during the first week and then gradually increase during follow‐up to 1 month [23]. In our cohort, FT3 and TSH increased across Day 1, 7, and 14, while severity‐related differences were most extensive on Day 7. Collectively, these findings support a time‐dependent and heterogeneous course of thyroid‐axis alterations after TBI.

Testosterone increased across the three scheduled time points in male patients, a temporal pattern that contrasts with the early decline reported in previous studies of acute severe TBI. Hohl et al. observed decreases in both LH and testosterone during approximately the first 3 days after severe TBI [29]. Wagner et al. also reported early suppression of the gonadal axis during the first post‐injury week, although testosterone trajectories varied among patients [30]. Barton et al. found that testosterone remained low during the first several weeks, with recovery beginning at approximately Week 6 in men without persistent hypogonadotropic hypogonadism [31]. In our cohort, although testosterone increased from Day 1 to 14, the median concentration remained below the lower laboratory reference limit at all three time points. Thus, the observed increase may represent partial recovery from early injury‐related suppression rather than an increase above the physiological range. Differences in injury severity and sampling schedules may partly account for the contrasting temporal patterns, because previous acute studies predominantly enrolled patients with severe TBI and focused on the first several days after injury, whereas our cohort included mild, moderate, and severe TBI and was assessed on Day 1, 7, and 14. Because no measurements were obtained between Day 1 and 7, an early decline during this interval followed by recovery cannot be excluded. The absence of a significant temporal change in LH further suggests that the observed testosterone increase may not be fully explained by parallel changes in pituitary LH secretion.

In the present study, day‐1 IL‐6, WBC, NEU, and PCT differed significantly across GCS‐defined severity groups. Previous clinical studies have similarly shown that circulating IL‐6 rises early after TBI and tends to be higher in patients with more severe injury, although its relationship with clinical outcome has varied across cohorts [3, 18]. On Day 1, IL‐6 was positively correlated with serum cortisol at 16:00, WBC and NEU were negatively correlated with T4, and PCT was negatively correlated with FT3. These exploratory correlations were limited to selected inflammatory marker–hormone pairs and varied across time points. Santarsieri et al. similarly found that associations between cerebrospinal fluid cortisol and inflammatory profiles after severe TBI differed according to cortisol trajectory and outcome group [32]. Our findings extend this literature by identifying same‐day associations between circulating inflammatory markers and both adrenal‐ and thyroid‐axis measures in a cohort spanning mild to severe TBI. Experimental studies have shown that IL‐6 can stimulate the adrenal axis independently of corticotropin‐releasing hormone during immune activation, providing one plausible mechanism by which early cytokine release may amplify stress‐endocrine responses after injury [33]. At the thyroid axis level, IL‐6 has also been shown to promote non‐thyroidal illness syndrome by reducing thyroxine activation and increasing thyroid hormone inactivation in human cells, offering a potential explanation for the association between inflammatory signaling and FT3 alterations after severe injury [34].

Although day‐1 IL‐6, NEU, PCT, T3, and FT3 differed between the favorable and unfavorable outcome groups, no significant associations with unfavorable 6‐month functional outcome were observed for IL‐6, WBC, NEU, PCT, day‐1 T3, or day‐1 FT3 after adjustment for admission GCS in separate Firth penalized logistic regression models. Therefore, the unadjusted group differences should be interpreted cautiously. The small number of unfavorable outcome events and the wide confidence intervals for day‐1 T3 and FT3 indicate limited precision and warrant cautious interpretation. Prior clinical studies have associated thyroid‐axis abnormalities with post‐traumatic neurological and functional outcomes, although evidence for these associations after adjustment for conventional severity indicators remains limited [11, 28, 35]. A potential relationship between thyroid‐axis abnormalities and functional outcomes is biologically plausible because thyroid hormones are involved in neuronal homeostasis, mitochondrial function, synaptic plasticity, and neural repair after brain injury [36, 37]. Larger studies with more outcome events and adjustment for additional potential confounders are needed to clarify these relationships.

Acute‐phase endocrine abnormalities after TBI may reflect transient responses to critical illness and do not necessarily predict persistent pituitary dysfunction, whereas some deficits may persist or become clinically apparent later during recovery [38, 39, 40, 41]. Current recommendations therefore prioritize recognition of clinically important adrenal insufficiency during the acute phase and advise reassessment of the adrenal, thyroid, and gonadal axes at approximately 3–6 months in patients with moderate‐to‐severe TBI or persistent symptoms [38, 40].

Several limitations should be acknowledged. This single‐center study had a relatively limited sample size and did not systematically capture detailed trauma severity scores or neurocritical care variables. Because dedicated pituitary MRI was not performed, the relationship between structural pituitary abnormalities and the observed endocrine changes was not evaluated. Blood pressure was assessed at a single baseline time point and therefore may not reflect hemodynamic changes during the acute clinical course. Hospital length of stay, GCS at discharge, and discharge disposition were not evaluated as study outcomes, which limited characterization of the early clinical course. The absence of extracranial trauma and non‐traumatic acute brain injury control groups limits attribution of the observed endocrine and inflammatory alterations to brain injury itself. Residual confounding from demographic, injury‐related, and acute management factors should also be considered. Moreover, the prospective observational design precludes causal inference regarding the relationships among endocrine alterations, inflammation, and clinical outcomes. Although multiple hormonal axes were evaluated, not all endocrine parameters were assessed, and dynamic endocrine changes beyond the acute phase were not fully captured. Inflammatory markers were measured only on Day 1, which precluded evaluation of their temporal changes during the acute phase. The inflammatory marker panel was limited in scope and did not allow comprehensive characterization of the systemic inflammatory response. Finally, although separate Firth penalized logistic regression models were used to adjust IL‐6, WBC, NEU, PCT, day‐1 T3, and day‐1 FT3 individually for admission GCS, the limited number of unfavorable outcome events did not permit reliable adjustment for additional potential confounders. Therefore, the outcome‐related analyses should be considered exploratory and require validation in larger multicenter cohorts.

## 5 Conclusions

Acute multi‐axis endocrine alterations in hospitalized patients after TBI were heterogeneous and time‐dependent, with prominent temporal changes involving cortisol, thyroid‐axis markers, and selected male gonadal hormones. Although day‐1 IL‐6, NEU, PCT, T3, and FT3 differed between the favorable and unfavorable outcome groups, no significant associations with unfavorable 6‐month functional outcome were observed for IL‐6, WBC, NEU, PCT, day‐1 T3, or day‐1 FT3 after adjustment for admission GCS in separate Firth models. Larger multicenter studies with more outcome events and adjustment for additional potential confounders are needed to clarify these relationships.

## Author Contributions

**Biao Xu** and **Peng Guo:** conceptualization, methodology, formal analysis, and writing original draft. **Zhenghao Wang**, **Jingchao Zhou**, and **Xingzhe Zhang:** investigation, data curation, software, and validation. **Rui Pan** and **Pengcheng Zhang:** visualization, formal analysis, and resources. **Zhanfeng Niu**, **Haibin Ma**, and **Qinghua Zhang:** conceptualization, supervision, project administration, funding acquisition, and writing – review and editing. All authors contributed to data collection, review, and editing. All authors have read and approved the final version of the manuscript.

## Funding

This work was supported by the Natural Science Foundation of Ningxia Province (Grant 2019AAC03224 and 2025AAC030802), the National Natural Science Foundation of China (Grant 82160266).

## Disclosure

Preprint Statement: An earlier version of this manuscript was posted on Research Square as a preprint entitled “Acute Multi‐Axis Endocrine Alterations After Traumatic Brain Injury: Associations With Injury Severity and 6‐Month Functional Outcome.” The preprint is available at: https://doi.org/10.21203/rs.3.rs‐9597854/v1.

## Ethics Statement

The protocol of this prospective observational cohort study was approved by the Ethics Committee of the General Hospital of Ningxia Medical University (Approval No. KYLL‐2024‐0014). Written informed consent was obtained from each participant or from a legally authorized representative before enrollment. The study was conducted in accordance with the Declaration of Helsinki.

## Conflicts of Interest

The authors declare no conflicts of interest.

## Supporting information


**Table S1: Supplementary** Reference ranges for hormone and inflammatory marker measurements.

**Table S2: Supplementary** Dynamic changes in hormone levels over time after traumatic brain injury.

**Table S3: Supplementary** Correlations between Day 1 inflammatory markers and core hormone levels at Day 1, 7, and 14.

**Table S4: Supplementary** Correlations between Day 1 inflammatory markers and sex‐hormone‐axis levels in male patients.

**Table S5: Supplementary** Comparison of sex‐hormone‐axis levels between male patients with favorable and unfavorable 6‐month outcomes.

## Data Availability

The datasets used or analyzed during the current study are available from the corresponding author on reasonable request.
