# Supplementary material for: Acute Endocrine Alterations After Traumatic Brain Injury and 6‐Month Functional Outcome
Source: Ann Clin Transl Neurol. 2026 Oct 29:10.1002/acn3.70570. Online ahead of print. doi: 10.1002/acn3.70570 (PMC13717412; doi:10.1002/acn3.70570)
Supplement: Supplementary file 1 [file ACN3-9999-0-s001.docx]

**Supplementary Table S4. Correlations between Day 1 inflammatory markers and sex-hormone-axis levels in male patients**

| **Hormone time point** | **Sex hormone** | **Inflammatory marker** | **Spearman r (95% CI)** | ***p* value** | **N** |
| --- | --- | --- | --- | --- | --- |
| Day 1 | LH | IL-6 | -0.232 (-0.578, 0.185) | 0.264 | 25 |
| Day 1 | LH | WBC | -0.098 (-0.332, 0.148) | 0.435 | 66 |
| Day 1 | LH | NEU | -0.049 (-0.288, 0.195) | 0.695 | 66 |
| Day 1 | LH | PCT | -0.075 (-0.408, 0.276) | 0.677 | 33 |
| Day 1 | FSH | IL-6 | -0.143 (-0.511, 0.269) | 0.495 | 25 |
| Day 1 | FSH | WBC | -0.152 (-0.381, 0.095) | 0.223 | 66 |
| Day 1 | FSH | NEU | -0.128 (-0.360, 0.119) | 0.306 | 66 |
| Day 1 | FSH | PCT | -0.016 (-0.357, 0.329) | 0.929 | 33 |
| Day 1 | PRL | IL-6 | 0.080 (-0.326, 0.461) | 0.705 | 25 |
| Day 1 | PRL | WBC | 0.168 (-0.079, 0.395) | 0.178 | 66 |
| Day 1 | PRL | NEU | 0.156 (-0.091, 0.385) | 0.211 | 66 |
| Day 1 | PRL | PCT | 0.125 (-0.230, 0.450) | 0.489 | 33 |
| Day 1 | TSTO | IL-6 | 0.034 (-0.366, 0.423) | 0.872 | 25 |
| Day 1 | TSTO | WBC | -0.298 (-0.508, -0.055) | 0.015 | 66 |
| Day 1 | TSTO | NEU | -0.278 (-0.491, -0.034) | 0.024 | 66 |
| Day 1 | TSTO | PCT | -0.248 (-0.549, 0.110) | 0.164 | 33 |
| Day 1 | E2 | IL-6 | -0.159 (-0.523, 0.254) | 0.447 | 25 |
| Day 1 | E2 | WBC | 0.088 (-0.158, 0.323) | 0.483 | 66 |
| Day 1 | E2 | NEU | 0.107 (-0.139, 0.341) | 0.390 | 66 |
| Day 1 | E2 | PCT | 0.105 (-0.248, 0.433) | 0.562 | 33 |
| Day 1 | PRGE | IL-6 | 0.277 (-0.141, 0.611) | 0.181 | 25 |
| Day 1 | PRGE | WBC | 0.278 (0.034, 0.491) | 0.024 | 66 |
| Day 1 | PRGE | NEU | 0.300 (0.057, 0.510) | 0.014 | 66 |
| Day 1 | PRGE | PCT | 0.098 (-0.255, 0.428) | 0.587 | 33 |
| Day 7 | LH | IL-6 | -0.037 (-0.442, 0.382) | 0.868 | 23 |
| Day 7 | LH | WBC | -0.314 (-0.550, -0.032) | 0.026 | 50 |
| Day 7 | LH | NEU | -0.231 (-0.482, 0.054) | 0.106 | 50 |
| Day 7 | LH | PCT | -0.323 (-0.618, 0.052) | 0.082 | 30 |
| Day 7 | FSH | IL-6 | -0.156 (-0.536, 0.276) | 0.477 | 23 |
| Day 7 | FSH | WBC | -0.514 (-0.703, -0.259) | <0.001 | 50 |
| Day 7 | FSH | NEU | -0.442 (-0.650, -0.174) | 0.001 | 50 |
| Day 7 | FSH | PCT | -0.086 (-0.433, 0.284) | 0.653 | 30 |
| Day 7 | PRL | IL-6 | -0.125 (-0.513, 0.304) | 0.568 | 23 |
| Day 7 | PRL | WBC | -0.140 (-0.404, 0.145) | 0.331 | 50 |
| Day 7 | PRL | NEU | -0.108 (-0.375, 0.177) | 0.457 | 50 |
| Day 7 | PRL | PCT | -0.328 (-0.622, 0.047) | 0.077 | 30 |
| Day 7 | TSTO | IL-6 | -0.215 (-0.580, 0.221) | 0.324 | 23 |
| Day 7 | TSTO | WBC | -0.556 (-0.733, -0.309) | <0.001 | 50 |
| Day 7 | TSTO | NEU | -0.521 (-0.707, -0.266) | <0.001 | 50 |
| Day 7 | TSTO | PCT | -0.462 (-0.715, -0.103) | 0.010 | 30 |
| Day 7 | E2 | IL-6 | -0.349 (-0.673, 0.087) | 0.103 | 23 |
| Day 7 | E2 | WBC | -0.379 (-0.601, -0.103) | 0.007 | 50 |
| Day 7 | E2 | NEU | -0.367 (-0.591, -0.089) | 0.009 | 50 |
| Day 7 | E2 | PCT | 0.009 (-0.352, 0.368) | 0.962 | 30 |
| Day 7 | PRGE | IL-6 | 0.173 (-0.261, 0.548) | 0.431 | 23 |
| Day 7 | PRGE | WBC | 0.333 (0.052, 0.564) | 0.018 | 50 |
| Day 7 | PRGE | NEU | 0.325 (0.043, 0.558) | 0.021 | 50 |
| Day 7 | PRGE | PCT | -0.045 (-0.399, 0.321) | 0.814 | 30 |
| Day 14 | LH | IL-6 | -0.393 (-0.763, 0.170) | 0.147 | 15 |
| Day 14 | LH | WBC | -0.174 (-0.528, 0.232) | 0.395 | 26 |
| Day 14 | LH | NEU | -0.100 (-0.470, 0.300) | 0.627 | 26 |
| Day 14 | LH | PCT | -0.264 (-0.646, 0.224) | 0.275 | 19 |
| Day 14 | FSH | IL-6 | 0.100 (-0.436, 0.583) | 0.723 | 15 |
| Day 14 | FSH | WBC | -0.174 (-0.528, 0.232) | 0.395 | 26 |
| Day 14 | FSH | NEU | -0.086 (-0.459, 0.313) | 0.677 | 26 |
| Day 14 | FSH | PCT | 0.077 (-0.391, 0.514) | 0.753 | 19 |
| Day 14 | PRL | IL-6 | 0.182 (-0.368, 0.638) | 0.516 | 15 |
| Day 14 | PRL | WBC | 0.349 (-0.057, 0.656) | 0.081 | 26 |
| Day 14 | PRL | NEU | 0.337 (-0.070, 0.647) | 0.093 | 26 |
| Day 14 | PRL | PCT | 0.280 (-0.209, 0.657) | 0.246 | 19 |
| Day 14 | TSTO | IL-6 | -0.246 (-0.678, 0.312) | 0.376 | 15 |
| Day 14 | TSTO | WBC | -0.576 (-0.800, -0.212) | 0.002 | 26 |
| Day 14 | TSTO | NEU | -0.523 (-0.768, -0.143) | 0.006 | 26 |
| Day 14 | TSTO | PCT | -0.395 (-0.729, 0.091) | 0.094 | 19 |
| Day 14 | E2 | IL-6 | -0.382 (-0.757, 0.181) | 0.160 | 15 |
| Day 14 | E2 | WBC | -0.605 (-0.816, -0.250) | 0.001 | 26 |
| Day 14 | E2 | NEU | -0.586 (-0.805, -0.225) | 0.002 | 26 |
| Day 14 | E2 | PCT | -0.373 (-0.716, 0.114) | 0.116 | 19 |
| Day 14 | PRGE | IL-6 | 0.041 (-0.482, 0.542) | 0.886 | 15 |
| Day 14 | PRGE | WBC | 0.042 (-0.351, 0.423) | 0.838 | 26 |
| Day 14 | PRGE | NEU | -0.009 (-0.395, 0.380) | 0.967 | 26 |
| Day 14 | PRGE | PCT | -0.198 (-0.602, 0.286) | 0.416 | 19 |

Note. Spearman rank correlations were calculated using pairwise complete observations, and analyses were restricted to male patients. Two-sided p values are reported. Given the exploratory nature of these analyses, *p* values were not adjusted for multiple testing. The 95% CIs were calculated using the Bonett–Wright Fisher z approximation. Inflammatory markers included IL-6, WBC, NEU, and PCT. LH, luteinizing hormone. FSH, follicle-stimulating hormone. PRL, prolactin. TSTO, testosterone. E2, estradiol. PRGE, progesterone. IL-6, interleukin-6. WBC, white blood cell count. NEU, neutrophil count. PCT, procalcitonin. CI, confidence interval.
