# Supplementary material for: Acute Endocrine Alterations After Traumatic Brain Injury and 6‐Month Functional Outcome
Source: Ann Clin Transl Neurol. 2026 Oct 29:10.1002/acn3.70570. Online ahead of print. doi: 10.1002/acn3.70570 (PMC13717412; doi:10.1002/acn3.70570)
Supplement: Supplementary file 2 [file ACN3-9999-0-s002.docx]

**Supplementary Table S1. Reference ranges for hormone and inflammatory marker measurements**

| **Hormone/Marker** | **Unit** | **Lower** | **Upper** |
| --- | --- | --- | --- |
| T4 | **μ**g/dL | 4.50 | 10.90 |
| T3 | ng/mL | 0.60 | 1.81 |
| FT4 | ng/dL | 0.89 | 1.76 |
| FT3 | pg/mL | 2.30 | 4.20 |
| TSH | **μ**IU/mL | 0.55 | 4.78 |
| hGH | ng/mL | 0.03 | 2.47 |
| ACTH | pg/mL | 7.20 | 63.30 |
| Cortisol | **μ**g/dL | 3.44 | 18.76 |
| LH | mIU/mL | 1.50 | 9.30 |
| FSH | mIU/mL | 1.40 | 18.10 |
| PRL | ng/mL | 2.10 | 17.70 |
| E2 | pg/mL | 0.00 | 39.80 |
| PRGE | ng/mL | 0.28 | 1.22 |
| TSTO | ng/dL | 187.72 | 684.19 |
| IL-6 | pg/mL | 0.00 | 7.00 |
| NEU | **×**10^9^/L | 1.80 | 6.30 |
| PCT | ng/mL | 0.00 | 0.09 |
| WBC | **×**10^9^/L | 3.50 | 9.50 |

Note. T4, thyroxine; T3, triiodothyronine; FT4, free thyroxine; FT3, free triiodothyronine; TSH, thyroid-stimulating hormone; hGH, human growth hormone; ACTH, adrenocorticotropic hormone; LH, luteinizing hormone; FSH, follicle-stimulating hormone; PRL, prolactin; E2, estradiol; PRGE, progesterone; TSTO, testosterone; IL-6, interleukin-6; NEU, neutrophil count; PCT, procalcitonin; WBC, white blood cell count.
