# Supplementary material for: Acute Endocrine Alterations After Traumatic Brain Injury and 6‐Month Functional Outcome
Source: Ann Clin Transl Neurol. 2026 Oct 29:10.1002/acn3.70570. Online ahead of print. doi: 10.1002/acn3.70570 (PMC13717412; doi:10.1002/acn3.70570)
Supplement: Supplementary file 3 [file ACN3-9999-0-s003.docx]

**Supplementary Table S3. Correlations between Day 1 inflammatory markers and core hormone levels at Days 1, 7, and 14**

| **Hormone time point** | **Hormone** | **Inflammatory marker** | **Spearman r (95% CI)** | ***p* value** | **N** |
| --- | --- | --- | --- | --- | --- |
| Day 1 | ACTH | IL-6 | 0.223 (-0.095, 0.500) | 0.161 | 41 |
| Day 1 | ACTH | WBC | -0.055 (-0.249, 0.143) | 0.583 | 100 |
| Day 1 | ACTH | NEU | -0.078 (-0.271, 0.121) | 0.439 | 100 |
| Day 1 | ACTH | PCT | 0.138 (-0.144, 0.400) | 0.334 | 51 |
| Day 1 | Cortisol at 08:00 | IL-6 | 0.257 (-0.060, 0.527) | 0.105 | 41 |
| Day 1 | Cortisol at 08:00 | WBC | 0.159 (-0.040, 0.346) | 0.114 | 100 |
| Day 1 | Cortisol at 08:00 | NEU | 0.131 (-0.068, 0.320) | 0.195 | 100 |
| Day 1 | Cortisol at 08:00 | PCT | -0.061 (-0.331, 0.219) | 0.669 | 51 |
| Day 1 | Cortisol at 16:00 | IL-6 | 0.498 (0.207, 0.708) | <0.001 | 41 |
| Day 1 | Cortisol at 16:00 | WBC | 0.065 (-0.133, 0.258) | 0.521 | 100 |
| Day 1 | Cortisol at 16:00 | NEU | 0.058 (-0.140, 0.252) | 0.568 | 100 |
| Day 1 | Cortisol at 16:00 | PCT | 0.201 (-0.082, 0.454) | 0.157 | 51 |
| Day 1 | T3 | IL-6 | 0.020 (-0.306, 0.342) | 0.908 | 37 |
| Day 1 | T3 | WBC | -0.072 (-0.270, 0.132) | 0.490 | 95 |
| Day 1 | T3 | NEU | -0.125 (-0.319, 0.079) | 0.226 | 95 |
| Day 1 | T3 | PCT | -0.202 (-0.465, 0.093) | 0.174 | 47 |
| Day 1 | T4 | IL-6 | 0.167 (-0.168, 0.468) | 0.324 | 37 |
| Day 1 | T4 | WBC | -0.311 (-0.486, -0.112) | 0.002 | 95 |
| Day 1 | T4 | NEU | -0.337 (-0.509, -0.140) | <0.001 | 95 |
| Day 1 | T4 | PCT | 0.109 (-0.185, 0.385) | 0.465 | 47 |
| Day 1 | FT3 | IL-6 | 0.027 (-0.300, 0.348) | 0.872 | 37 |
| Day 1 | FT3 | WBC | 0.029 (-0.174, 0.229) | 0.777 | 95 |
| Day 1 | FT3 | NEU | 0.008 (-0.194, 0.209) | 0.935 | 95 |
| Day 1 | FT3 | PCT | -0.353 (-0.587, -0.064) | 0.015 | 47 |
| Day 1 | FT4 | IL-6 | 0.251 (-0.085, 0.536) | 0.133 | 37 |
| Day 1 | FT4 | WBC | -0.112 (-0.307, 0.092) | 0.282 | 95 |
| Day 1 | FT4 | NEU | -0.123 (-0.317, 0.081) | 0.234 | 95 |
| Day 1 | FT4 | PCT | 0.047 (-0.244, 0.330) | 0.752 | 47 |
| Day 1 | TSH | IL-6 | -0.059 (-0.376, 0.270) | 0.727 | 37 |
| Day 1 | TSH | WBC | -0.178 (-0.368, 0.026) | 0.084 | 95 |
| Day 1 | TSH | NEU | -0.211 (-0.398, -0.008) | 0.040 | 95 |
| Day 1 | TSH | PCT | 0.005 (-0.283, 0.292) | 0.975 | 47 |
| Day 1 | hGH | IL-6 | -0.012 (-0.335, 0.313) | 0.946 | 37 |
| Day 1 | hGH | WBC | 0.051 (-0.152, 0.250) | 0.623 | 95 |
| Day 1 | hGH | NEU | 0.059 (-0.144, 0.258) | 0.567 | 95 |
| Day 1 | hGH | PCT | 0.044 (-0.246, 0.327) | 0.769 | 47 |
| Day 7 | ACTH | IL-6 | -0.016 (-0.352, 0.324) | 0.930 | 34 |
| Day 7 | ACTH | WBC | -0.261 (-0.472, -0.022) | 0.030 | 69 |
| Day 7 | ACTH | NEU | -0.251 (-0.463, -0.011) | 0.037 | 69 |
| Day 7 | ACTH | PCT | -0.177 (-0.462, 0.141) | 0.268 | 41 |
| Day 7 | Cortisol at 08:00 | IL-6 | 0.277 (-0.074, 0.567) | 0.113 | 34 |
| Day 7 | Cortisol at 08:00 | WBC | 0.210 (-0.031, 0.428) | 0.083 | 69 |
| Day 7 | Cortisol at 08:00 | NEU | 0.145 (-0.096, 0.370) | 0.233 | 69 |
| Day 7 | Cortisol at 08:00 | PCT | 0.066 (-0.247, 0.367) | 0.681 | 41 |
| Day 7 | Cortisol at 16:00 | IL-6 | 0.378 (0.033, 0.642) | 0.027 | 34 |
| Day 7 | Cortisol at 16:00 | WBC | -0.005 (-0.241, 0.232) | 0.967 | 69 |
| Day 7 | Cortisol at 16:00 | NEU | 0.024 (-0.214, 0.259) | 0.842 | 69 |
| Day 7 | Cortisol at 16:00 | PCT | 0.190 (-0.128, 0.472) | 0.234 | 41 |
| Day 7 | T3 | IL-6 | -0.280 (-0.569, 0.071) | 0.109 | 34 |
| Day 7 | T3 | WBC | -0.305 (-0.509, -0.068) | 0.011 | 69 |
| Day 7 | T3 | NEU | -0.312 (-0.515, -0.076) | 0.009 | 69 |
| Day 7 | T3 | PCT | -0.217 (-0.495, 0.101) | 0.172 | 41 |
| Day 7 | T4 | IL-6 | -0.451 (-0.694, -0.116) | 0.007 | 34 |
| Day 7 | T4 | WBC | -0.450 (-0.628, -0.227) | <0.001 | 69 |
| Day 7 | T4 | NEU | -0.440 (-0.620, -0.216) | <0.001 | 69 |
| Day 7 | T4 | PCT | -0.226 (-0.502, 0.092) | 0.155 | 41 |
| Day 7 | FT3 | IL-6 | -0.278 (-0.568, 0.073) | 0.112 | 34 |
| Day 7 | FT3 | WBC | -0.248 (-0.461, -0.008) | 0.040 | 69 |
| Day 7 | FT3 | NEU | -0.236 (-0.450, 0.004) | 0.051 | 69 |
| Day 7 | FT3 | PCT | -0.282 (-0.547, 0.034) | 0.075 | 41 |
| Day 7 | FT4 | IL-6 | -0.203 (-0.509, 0.149) | 0.250 | 34 |
| Day 7 | FT4 | WBC | -0.291 (-0.497, -0.053) | 0.015 | 69 |
| Day 7 | FT4 | NEU | -0.258 (-0.469, -0.019) | 0.032 | 69 |
| Day 7 | FT4 | PCT | -0.246 (-0.518, 0.071) | 0.121 | 41 |
| Day 7 | TSH | IL-6 | -0.451 (-0.694, -0.116) | 0.007 | 34 |
| Day 7 | TSH | WBC | -0.214 (-0.431, 0.027) | 0.077 | 69 |
| Day 7 | TSH | NEU | -0.290 (-0.497, -0.052) | 0.016 | 69 |
| Day 7 | TSH | PCT | -0.246 (-0.518, 0.071) | 0.121 | 41 |
| Day 7 | hGH | IL-6 | -0.048 (-0.380, 0.295) | 0.789 | 34 |
| Day 7 | hGH | WBC | -0.028 (-0.263, 0.210) | 0.822 | 69 |
| Day 7 | hGH | NEU | 0.006 (-0.231, 0.242) | 0.959 | 69 |
| Day 7 | hGH | PCT | 0.109 (-0.206, 0.404) | 0.498 | 41 |
| Day 14 | ACTH | IL-6 | -0.065 (-0.484, 0.378) | 0.780 | 21 |
| Day 14 | ACTH | WBC | -0.141 (-0.459, 0.209) | 0.428 | 34 |
| Day 14 | ACTH | NEU | -0.130 (-0.450, 0.219) | 0.463 | 34 |
| Day 14 | ACTH | PCT | -0.106 (-0.475, 0.294) | 0.606 | 26 |
| Day 14 | Cortisol at 08:00 | IL-6 | -0.138 (-0.530, 0.303) | 0.539 | 22 |
| Day 14 | Cortisol at 08:00 | WBC | -0.258 (-0.545, 0.083) | 0.128 | 36 |
| Day 14 | Cortisol at 08:00 | NEU | -0.232 (-0.524, 0.109) | 0.173 | 36 |
| Day 14 | Cortisol at 08:00 | PCT | -0.119 (-0.479, 0.275) | 0.553 | 27 |
| Day 14 | Cortisol at 16:00 | IL-6 | -0.111 (-0.510, 0.327) | 0.623 | 22 |
| Day 14 | Cortisol at 16:00 | WBC | -0.117 (-0.430, 0.221) | 0.496 | 36 |
| Day 14 | Cortisol at 16:00 | NEU | -0.118 (-0.431, 0.220) | 0.492 | 36 |
| Day 14 | Cortisol at 16:00 | PCT | -0.256 (-0.584, 0.144) | 0.197 | 27 |
| Day 14 | T3 | IL-6 | -0.031 (-0.457, 0.406) | 0.895 | 21 |
| Day 14 | T3 | WBC | -0.256 (-0.547, 0.090) | 0.137 | 35 |
| Day 14 | T3 | NEU | -0.342 (-0.612, 0.000) | 0.044 | 35 |
| Day 14 | T3 | PCT | -0.104 (-0.473, 0.296) | 0.612 | 26 |
| Day 14 | T4 | IL-6 | -0.275 (-0.637, 0.186) | 0.228 | 21 |
| Day 14 | T4 | WBC | -0.392 (-0.649, -0.055) | 0.020 | 35 |
| Day 14 | T4 | NEU | -0.433 (-0.678, -0.101) | 0.009 | 35 |
| Day 14 | T4 | PCT | -0.328 (-0.641, 0.079) | 0.102 | 26 |
| Day 14 | FT3 | IL-6 | 0.220 (-0.239, 0.599) | 0.339 | 21 |
| Day 14 | FT3 | WBC | -0.138 (-0.452, 0.206) | 0.429 | 35 |
| Day 14 | FT3 | NEU | -0.215 (-0.515, 0.131) | 0.216 | 35 |
| Day 14 | FT3 | PCT | -0.125 (-0.490, 0.277) | 0.542 | 26 |
| Day 14 | FT4 | IL-6 | 0.247 (-0.213, 0.618) | 0.281 | 21 |
| Day 14 | FT4 | WBC | -0.135 (-0.449, 0.209) | 0.440 | 35 |
| Day 14 | FT4 | NEU | -0.150 (-0.462, 0.195) | 0.390 | 35 |
| Day 14 | FT4 | PCT | -0.136 (-0.499, 0.267) | 0.508 | 26 |
| Day 14 | TSH | IL-6 | 0.042 (-0.397, 0.465) | 0.858 | 21 |
| Day 14 | TSH | WBC | 0.061 (-0.278, 0.387) | 0.726 | 35 |
| Day 14 | TSH | NEU | -0.088 (-0.410, 0.253) | 0.614 | 35 |
| Day 14 | TSH | PCT | 0.006 (-0.382, 0.392) | 0.975 | 26 |
| Day 14 | hGH | IL-6 | 0.036 (-0.402, 0.461) | 0.878 | 21 |
| Day 14 | hGH | WBC | 0.234 (-0.112, 0.530) | 0.177 | 35 |
| Day 14 | hGH | NEU | 0.336 (-0.007, 0.608) | 0.048 | 35 |
| Day 14 | hGH | PCT | -0.170 (-0.525, 0.235) | 0.406 | 26 |

Note. Spearman rank correlations were calculated using pairwise complete observations. Two-sided p values are reported. Given the exploratory nature of these analyses, *p* values were not adjusted for multiple testing. The 95% CIs were calculated using the Bonett–Wright Fisher z approximation. Inflammatory markers included IL-6, WBC, NEU, and PCT. IL-6, interleukin-6. ACTH, adrenocorticotropic hormone. WBC, white blood cell count. NEU, neutrophil count. PCT, procalcitonin. hGH, human growth hormone. CI, confidence interval.
