# Supplementary material for: Acute Endocrine Alterations After Traumatic Brain Injury and 6‐Month Functional Outcome
Source: Ann Clin Transl Neurol. 2026 Oct 29:10.1002/acn3.70570. Online ahead of print. doi: 10.1002/acn3.70570 (PMC13717412; doi:10.1002/acn3.70570)
Supplement: Supplementary file 4 [file ACN3-9999-0-s004.docx]

**Supplementary Table S5. Comparison of sex-hormone-axis levels between male patients with favorable and unfavorable 6-month outcomes**

| **Time point** | **Variable** | **Favorable outcome** | **Unfavorable outcome** | ***p* value** | **Effect size (95% CI)** |
| --- | --- | --- | --- | --- | --- |
| Day 1 | LH | 3.94 (2.95, 5.45) | 3.20 (2.42, 4.99) | 0.258 | 0.210 (-0.150, 0.520) |
| Day 1 | FSH | 5.98 (4.35, 8.01) | 4.52 (2.00, 9.35) | 0.383 | 0.162 (-0.198, 0.483) |
| Day 1 | PRL | 9.06 (6.91, 12.09) | 9.35 (7.28, 10.77) | 0.967 | -0.008 (-0.355, 0.342) |
| Day 1 | TSTO | 148.04 (62.65, 244.47) | 55.75 (24.46, 232.87) | 0.070 | 0.336 (-0.014, 0.613) |
| Day 1 | E2 | 29.45 (23.38, 37.23) | 26.32 (21.66, 35.76) | 0.648 | 0.085 (-0.272, 0.421) |
| Day 1 | PRGE | 0.52 (0.30, 0.80) | 0.37 (0.23, 0.67) | 0.298 | 0.193 (-0.167, 0.507) |
| Day 7 | LH | 4.21 (3.01, 6.18) | 3.80 (2.25, 5.71) | 0.373 | 0.177 (-0.208, 0.515) |
| Day 7 | FSH | 5.89 (3.59, 7.97) | 4.15 (1.97, 9.31) | 0.271 | 0.219 (-0.166, 0.546) |
| Day 7 | PRL | 12.42 (9.74, 14.64) | 9.19 (2.78, 13.52) | 0.099 | 0.329 (-0.049, 0.624) |
| Day 7 | TSTO | 166.06 (71.31, 327.42) | 51.31 (27.68, 179.50) | 0.027 | 0.441 (0.083, 0.698) |
| Day 7 | E2 | 28.31 (21.76, 37.47) | 26.42 (17.82, 33.92) | 0.699 | 0.077 (-0.303, 0.436) |
| Day 7 | PRGE | 0.31 (0.11, 0.43) | 0.11 (0.11, 0.34) | 0.189 | 0.252 (-0.132, 0.570) |
| Day 14 | LH | 3.94 (2.85, 5.29) | 4.29 (2.79, 8.12) | 0.732 | -0.081 (-0.498, 0.366) |
| Day 14 | FSH | 5.69 (3.65, 8.32) | 5.20 (3.88, 8.61) | 0.916 | 0.025 (-0.414, 0.454) |
| Day 14 | PRL | 12.17 (9.93, 19.57) | 12.27 (4.01, 14.67) | 0.635 | 0.113 (-0.338, 0.521) |
| Day 14 | TSTO | 193.37 (112.74, 373.73) | 144.30 (47.17, 280.13) | 0.206 | 0.300 (-0.154, 0.649) |
| Day 14 | E2 | 28.31 (20.33, 36.29) | 24.30 (18.65, 34.69) | 0.510 | 0.156 (-0.298, 0.553) |
| Day 14 | PRGE | 0.33 (0.11, 0.50) | 0.11 (0.11, 0.36) | 0.120 | 0.350 (-0.099, 0.681) |

Note. Data are presented as median (P25, P75). Analyses were restricted to male patients. Comparisons were based on available samples for each variable. *P* values were calculated using the Mann-Whitney U test. Effect sizes are reported as rank-biserial r with 95% CIs; positive values indicate higher levels in the favorable outcome group. LH, luteinizing hormone; FSH, follicle-stimulating hormone; PRL, prolactin; TSTO, testosterone; E2, estradiol; PRGE, progesterone. CI, confidence interval.
