# Supplementary material for: Acute Endocrine Alterations After Traumatic Brain Injury and 6‐Month Functional Outcome
Source: Ann Clin Transl Neurol. 2026 Oct 29:10.1002/acn3.70570. Online ahead of print. doi: 10.1002/acn3.70570 (PMC13717412; doi:10.1002/acn3.70570)
Supplement: Supplementary file 5 [file ACN3-9999-0-s005.docx]

**Supplementary Table S2. Dynamic changes in hormone levels over time after traumatic brain injury**

| **Hormone** | **Day 1** | **Day 7** | **Day 14** | ***p* value** | **Effect size (95% CI)** |
| --- | --- | --- | --- | --- | --- |
| **HPA axis** |  |  |  |  |  |
| ACTH | 29.43 (14.94, 49.96) | 35.27 (23.80, 50.89) | 33.16 (19.27, 45.58) | 0.812 | W = 0.004 (0.001, 0.076) |
| Cortisol at 08:00 | 29.16 (22.48, 38.78) | 19.97 (16.88, 25.00)* | 19.42 (15.45, 25.98)* | <0.001 | W = 0.205 (0.085, 0.388) Day 7 vs Day 1: r = −0.762 (−0.844, −0.646); Day 14 vs Day 1: r = −0.660 (−0.794, −0.466) |
| Cortisol at 16:00 | 22.93 (16.45, 33.07) | 15.59 (11.37, 21.27)* | 15.95 (9.77, 19.97)* | <0.001 | W = 0.239 (0.126, 0.408) Day 7 vs Day 1: r = −0.690 (−0.794, −0.548); Day 14 vs Day 1: r = −0.792 (−0.877, −0.657) |
| **Thyroid axis** |  |  |  |  |  |
| T3 | 0.75 (0.64, 0.88) | 0.85 (0.60, 1.01)* | 0.90 (0.70, 1.06)^#^ | 0.045 | W = 0.103 (0.018, 0.308) Day 7 vs Day 1: r = 0.350 (0.083, 0.569); Day 14 vs Day 7: r = 0.594 (0.294, 0.787) |
| T4 | 6.80 (5.50, 8.20) | 7.80 (5.80, 9.50) | 7.40 (5.90, 9.40) | 0.659 | W = 0.014 (0.001, 0.160) |
| FT3 | 2.47 (2.14, 2.90) | 2.74 (2.35, 3.22)* | 3.05 (2.26, 3.32)*,^#^ | 0.007 | W = 0.168 (0.043, 0.404) Day 7 vs Day 1: r = 0.463 (0.215, 0.654); Day 14 vs Day 1: r = 0.587 (0.257, 0.794); Day 14 vs Day 7: r = 0.690 (0.437, 0.842) |
| FT4 | 1.12 (1.01, 1.30) | 1.20 (1.05, 1.35) | 1.17 (0.98, 1.35) | 0.506 | W = 0.023 (0.002, 0.181) |
| TSH | 0.571 (0.300, 0.963) | 1.411 (0.625, 2.658)* | 2.031 (1.190, 2.972)*,^#^ | <0.001 | W = 0.303 (0.138, 0.541) Day 7 vs Day 1: r = 0.718 (0.553, 0.829); Day 14 vs Day 1: r = 0.905 (0.797, 0.957); Day 14 vs Day 7: r = 0.543 (0.224, 0.757) |
| **Sex-hormone axis** |  |  |  |  |  |
| LHᵃ | 3.87 (2.74, 5.28) | 4.16 (2.88, 6.04) | 3.94 (3.01, 5.76) | 0.293 | W = 0.056 (0.006, 0.287) |
| FSHᵃ | 5.76 (4.01, 8.04) | 5.59 (3.20, 8.13) | 5.69 (3.89, 7.77) | 0.108 | W = 0.101 (0.014, 0.349) |
| PRLᵃ | 9.06 (7.01, 12.09) | 12.00 (8.76, 14.33) | 12.17 (9.89, 16.46) | 0.062 | W = 0.126 (0.014, 0.405) |
| TSTOᵃ | 118.94 (58.17, 244.47) | 148.47 (52.30, 287.64)* | 160.05 (96.60, 315.87)*,^#^ | 0.001 | W = 0.306 (0.101, 0.620) Day 7 vs Day 1: r = 0.410 (0.103, 0.645); Day 14 vs Day 1: r = 0.692 (0.357, 0.869); Day 14 vs Day 7: r = 0.795 (0.568, 0.909) |
| E2ᵃ | 28.28 (23.20, 36.56) | 27.95 (21.63, 36.79) | 27.41 (20.37, 34.79) | 1.000 | W = 0.000 (0.000, 0.188) |
| PRGEᵃ | 0.495 (0.278, 0.785) | 0.290 (0.110, 0.403)* | 0.225 (0.110, 0.393)* | <0.001 | W = 0.666 (0.513, 0.820) Day 7 vs Day 1: r = −0.980 (−0.990, −0.962); Day 14 vs Day 1: r = −0.957 (−0.983, −0.892) |
| **Growth hormone** |  |  |  |  |  |
| hGH | 0.615 (0.381, 1.590) | 0.875 (0.388, 1.515) | 1.040 (0.402, 1.910) | 0.177 | W = 0.058 (0.008, 0.243) |

Note. Data are presented as median (P25, P75) based on available samples at each time point. *P* values were calculated using the Friedman test among patients with complete measurements at all three time points for each hormone. Post hoc pairwise comparisons were performed using the Wilcoxon signed-rank test with Bonferroni correction when the Friedman test was significant. **p* < 0.017 vs Day 1 after Bonferroni correction; ^#^*p* < 0.017 vs Day 7 after Bonferroni correction. ᵃLH, FSH, PRL, TSTO, E2, and PRGE were analyzed in male patients only. Kendall’s W is reported for omnibus comparisons, with 95% CIs estimated using 5,000 bootstrap resamples. Paired rank-biserial r with 95% CIs is reported for significant post hoc comparisons. Positive paired rank-biserial r values indicate higher levels at the first named time point. CI, confidence interval.
